# Neurogenic inducers inhibit the proliferation of pancreatic cancer by promoting tumor cell transdifferentiation

**DOI:** 10.1186/s13046-025-03563-9

**Published:** 2025-11-12

**Authors:** Duancheng Guo, Saimeng Shi, Longyun Ye, Mengdi Yang, Wenxia Peng, Jianhui Yang, Ji Xu, Qinglin Fei, Hao Li, Kaizhou Jin, Xichun Hu, Weiding Wu

**Affiliations:** 1 Department of Pancreatic Surgery, Fudan University Shanghai Cancer Center, Shanghai, 200032 China; 2 Department of Oncology, Shanghai Medical College, Fudan University, Shanghai, 200032 China; 3 Shanghai Pancreatic Cancer Institute, Shanghai, 200032 China; 4 Pancreatic Cancer Institute, Fudan University, Shanghai, 200032 China; 5 Department of Medical Oncology, Fudan University Shanghai Cancer Center, Shanghai, 200032 China; 6 State Key Laboratory of Bioreactor Engineering, Shanghai Key Laboratory of New Drug Design, School of Pharmacy, East China University of Science and Technology, Shanghai, 200237 China; 7 Department of Oncology and Hematology, Zhoupu Hospital, Pudong New Area, Shanghai, 201318 China

**Keywords:** Pancreatic cancer, NeuroD1, Transdifferentiation, Neurogenic inducers, Differentiation therapy

## Abstract

**Background:**

Tumor cell differentiation is a critical determinant of malignancy and clinical treatment selection. Pancreatic ductal adenocarcinoma (PDAC), a poorly differentiated and highly aggressive tumor, has a poor prognosis, whereas well-differentiated tumors often correlate with better outcomes. The mechanisms underlying differentiation and its therapeutic potential remain unclear.

**Objectives:**

This study aims to investigate whether inducing transdifferentiation in pancreatic cancer cells can reduce malignancy, focusing on the role of the transcription factor NeuroD1 and its regulatory pathways.

**Methods:**

We analyzed single-cell RNA-seq data from the GEO database to identify differentiation-associated genes. NeuroD1 was overexpressed in PDAC cells to assess its effects on transdifferentiation and proliferation. Drug screening and molecular docking were performed to identify differentiation-inducing compounds. RNA sequencing, coimmunoprecipitation, and mass spectrometry were used to identify NeuroD1-interacting proteins. Cell/patient-derived xenograft mouse models are utilized for in vivo experiments and compound efficacy testing.

**Results:**

Highly differentiated tumor cells exhibited elevated NeuroD1 expression. NeuroD1 overexpression promoted neuronal transdifferentiation and suppressed proliferation. Neuropathiazol, a neurogenic inducer, was found to bind MET and upregulate NeuroD1 via the PI3K/Akt pathway, enhancing transdifferentiation and inhibiting tumor growth. Neurog3 was identified as a functional partner of NeuroD1.

**Conclusion:**

Our findings demonstrate that pancreatic cancer cells can be induced to transdifferentiate through NeuroD1 activation or pharmacological induction, suggesting a potential therapeutic strategy to mitigate malignancy by reprogramming tumor cells into less aggressive states.

**Supplementary Information:**

The online version contains supplementary material available at 10.1186/s13046-025-03563-9.

## Introduction

Pancreatic cancer is an extremely aggressive malignancy of the digestive system. According to the latest data from *Global Cancer Statistics* published by the International Agency for Research, pancreatic cancer has emerged as the sixth leading cause of cancer-related mortality globally [1]. Its severity is further highlighted by the data published by the 2024 U.S. Cancer Statistics, in which it is ranked as the third leading cause of cancer-related death in the United States [2]. Epidemiological studies underscore the increasing public health significance of pancreatic cancer and show that its incidence is increasing annually. Approximately 90% of pancreatic cancers are pancreatic ductal adenocarcinomas (PDAC) [3]. At present, surgery is the sole treatment modality capable of curing pancreatic cancer [4]. However, due to the advanced stage at which the disease often presents, only 15–20% of patients are deemed eligible for surgical intervention at the time of diagnosis [5]. Chemotherapy remains the primary adjuvant treatment for all stages of pancreatic cancer, offering significant short-term improvements in patient prognosis. However, its long-term effectiveness in extending survival is limited by the development of drug resistance [6, 7]. Therefore, improving therapeutic efficacy and reducing treatment toxicity are important goals of current clinical trials and basic research on pancreatic cancer.

Tumor differentiation is the process occurring during tumor cell growth and development where these cells display morphological and functional features that are distinctive from those of normal tissue cells [8]. The degree of tumor cell differentiation is a key factor in determining tumor malignancy and serves as an important basis for selecting treatment strategies [9, 10]. PDAC originates from epithelial cells and is usually composed of poorly differentiated tumor cells, characterized by high malignancy and poor patient prognosis, whereas patients with highly differentiated tumors generally have better prognoses than those with poorly differentiated tumors [11, 12]. The capacity to modulate the degree of tumor cell differentiation holds immense significance for clinical tumor management and patient prognosis. This regulatory potential not only impacts the efficacy of anti-cancer interventions but also exerts a pivotal and decisive influence on predicting long-term survival outcomes. The mechanisms by which malignant tumor cells are reverted to normal or normal-like cell differentiation through gene editing or differentiation-inducing agents have recently become a focus of research [13–15]. Research has demonstrated that cancer-associated fibroblasts are able to induce the conversion of acinar cells to cells with a ductal phenotype [16]. Similarly, beta cells can be induced to transdifferentiate via neurogenic differentiation factor 1 (NeuroD1) [17]. In light of this, inducing the transdifferentiation of poorly differentiated pancreatic cancer cells into well-differentiated cells or into cells with normal morphology is anticipated to reduce the malignancy of pancreatic cancer and offers new hope for treatment.

The differentiation of normal cells or tumor cells is usually driven by specific transcription factors [18, 19]. Our study, which leverages bioinformatics analysis of data from the TCGA and GEO databases and validation through patient tissue samples, identified that NeuroD1 regulates the degree of differentiation of pancreatic cancer cells, acts as a protective factor for pancreatic cancer, and is associated with a better prognosis. NeuroD1 is a member of the NeuroD family, which encodes basic helix-loop-helix (bHLH) transcription factors. It plays a crucial role in inducing neuronal differentiation and regulating pancreatic development. The function of NeuroD1 in neuronal differentiation has been extensively documented. Notably, NeuroD1 has been shown to cause direct neuronal transformation of mouse microglia both in vitro and in vivo [20]. Following ischemic brain injury, NeuroD1 has the capacity to facilitate the in situ transformation of astrocytes into neurons, leading to the regeneration of a substantial number of new functional neurons [21]. In tumors, NeuroD1 has demonstrated ability to induce the transdifferentiation of medulloblastoma cells into mature neurons [22]. This process effectively and permanently eliminates the proliferative capacity and the tumorigenic potential of medulloblastoma cells, rendering them incapable of contributing to tumor growth. NeuroD1 was regarded as a promising inducing differentiation factor and therapeutic target for the treatment of medulloblastoma. However, previous studies on NeuroD1-induced transdifferentiation have been primarily focused on scenarios of neurological injury repair or neurogenic tumors. To date, no research has been reported on the role of NeuroD1 in the initiation and progression of pancreatic cancer. Existing studies have demonstrated that NeuroD1 can influence the fate determination of endocrine cells during pancreatic development [17, 23]. Based on this evidence, our study aimed to screen and identify key transcription factors (such as NeuroD1) that regulate the induction of transdifferentiation in pancreatic cancer cells. This represents the first time that the NeuroD1-mediated transdifferentiation mechanism has been introduced into solid tumors of the digestive system, breaking the previous limitation to the context of “neurogenesis/neurogenic tumors”. Through a series of in vitro and in vivo experiments, our studies have demonstrated that the overexpression of NeuroD1 facilitates the transdifferentiation of pancreatic cancer cells into neuron-like cells and that it inhibits tumor cell proliferation and progression both in vivo and in vitro. Using drug screening and computer simulations, we found that the neuronal inducer Neuropathiazol upregulates NeuroD1 expression by binding to the MET protein and inhibiting the activation of the PI3K/Akt signaling pathway. Further RNA sequencing and protein mass spectrometry identified Neurogenin 3 (Neurog3) as a protein that binds to NeuroD1. Consistent with this, Neurog3 was shown to inhibit tumor cell proliferation and to promote the neuronal transdifferentiation of tumor cells both in vivo and in vitro. Our research identifies NeuroD1 as a potential therapeutic target for pancreatic cancer and suggests that pancreatic tumor cells can be induced to revert to a normal or a normal-like state through gene editing or through the use of agents that induce cell differentiation, suggesting a new direction for the treatment of pancreatic cancer.

## Materials and methods

### Patients and specimens

A total of 240 diagnosed pancreatic cancer tissue samples were collected from patients who underwent surgical resection at Fudan University Shanghai Cancer Center (FUSCC) between January 2011 and December 2014. Patients who had received neoadjuvant therapy, or had inflammatory diseases or active infections, were excluded from the study. The data evaluated included patient demographics (sex and age), tumor characteristics (location, diameter, and CA199 concentration), TNM stage, and tumor grade. Tumor staging was determined according to the American Joint Committee on Cancer TNM classification (8th edition). Overall survival (OS) was defined as the time interval from the date of surgery to the date of death. This study was approved by the ethics committee at FUSCC (approval number: 050432-4-2307E), and all patients provided written informed consent in accordance with the principles of the Declaration of Helsinki.

### Cell culture

Human embryonic kidney 293 T cells (HEK293T) and several human pancreatic cancer cell lines, including SW1990, BxPC-3, Mia-PaCa-2, Capan-1, CFPAC-1, Asp3, and Panc-1, were purchased from Cell Bank, Type Culture Collection, Chinese Academy of Sciences. Additionally, the human neuroblastoma cell line CHP134, which was isolated from a patient at The Children’s Hospital of Philadelphia (Philadelphia, PA, USA), was purchased from the European Collection of Authenticated Cell Cultures (Public Health England, Salisbury, UK). All human cell lines have been authenticated using short tandem repeat (STR) profiling and have been tested to ensure they are free of mycoplasma contamination (40601ES20, Yeasen).

The cells were cultured in a humidified incubator at 37 °C with 5% CO₂. BxPC-3 cells were maintained in RPMI-1640 medium supplemented with 10% fetal bovine serum (FBS). Capan-1 and CFPAC-1 cells were cultured in IMDM medium containing 10% FBS. CHP134 cells were grown in RPMI-1640 medium supplemented with 10% FBS and 2 mM glutamine. The remaining cell lines were cultured in high-glucose DMEM medium containing 10% FBS.

### Lentivirus package and luciferase reporter assay

The overexpression plasmid was constructed by inserting the cDNA encoding NeuroD1 and Neurog3 genes into the CMV-MCS-EF1α-Puro vector (Addgene plasmid 72265). To generate lentiviral particles, HEK293T cells were cultured to 90% confluence in a 10 cm² dish and prepared for transfection. A total of 3 µg of the target plasmid DNA, along with 9 µg of psPAX2 and 9 µg of pMD2.G, was mixed with Lipofectamine™ 3000 reagent (L3000075, Thermo Fisher, Carlsbad, CA, USA) and diluted in Opti-MEM medium at room temperature for 20 min before being added to the cell culture medium. After incubation at 37 °C for 8 h, the cells were refreshed with 15 mL of complete medium, and the supernatant enriched with lentiviral particles was collected after 48 h.

The promoter sequence of the NeuroD1 gene (supplementary material Table S2) was synthesized and inserted into the pGL3-Basic vector (Tsingke Biotechnology, China). HEK293T cells were cultured to approximately 70% confluence in a 10 cm² dish and transfected with 5 µg of the promoter DNA using Lipofectamine™ 3000 reagent, followed by continued culture for 48 h. The transfected cells were then seeded into 96-well plates, treated with PBS and various compounds for 48 h, and the fluorescence intensity was measured at 560 nm using the Dual Luciferase Reporter Assay Kit (DLDL101-01, Vazyme, China).

### Animal model and drug treatment experiment

All mice were purchased from the Laboratory Animal Center of FUSCC and bred in a specific pathogen-free facility. The LSL-KRas^G12D/+^-p53^R172H/+^-Pdx1-Cre (KPC) transgenic mice were obtained from Gempharmatech Co., Ltd., Jiangsu, China. Genotyping was performed following the company’s recommended standard protocol, and KPC transgenic mice developed PDAC after 8 weeks of age. Murine pancreatic cancer cells were isolated from spontaneously arising tumors in KPC transgenic mice. Tumor tissues were removed from the pancreas and digested in 5 mL of RPMI-1640 basic medium containing 2 mg/mL Collagenase IV (Roche) and 200 U/mL DNase (Sigma) for 30 min at 37 °C. The digestion was terminated by adding RPMI-1640 complete medium with 10% FBS to obtain a single-cell suspension. These cells were used to generate a pancreas orthotopic transplantation KPC model.

For basic research, subcutaneous tumor models were established. Briefly, female BALB/c-nude mice (4–6 weeks old) were subcutaneously injected with approximately 5 × 10^6^ Panc-1 or SW1990 cells in 100 µL PBS on the left flank. NSG mice were subcutaneously implanted with PDAC specimens according to our center’s previously described procedures [24]. Surgically resected PDAC samples were cut into equal blocks of approximately 10 mm^3^ and transplanted subcutaneously into the flanks of female NSG mice (6–8 weeks old). Tumor volume was measured and calculated using the formula (W^2^×L)/2 (*W*, width; *L*, length). When the tumor volume reached approximately 200 mm^3^, the mice were randomly assigned to different treatment groups. For in vivo treatment experiments, the following compounds were used at the indicated doses: Neuropathiazol (15 mg/kg or 50 mg/kg), gemcitabine (20 mg/kg), cisplatin (10 mg/kg), and paclitaxel (15 mg/kg) [25]. These compounds were dissolved in 3% DMSO in MCT (0.5% methyl cellulose containing 0.2% Tween-80, Solarbio). Tumor-bearing mice were injected intraperitoneally with the compounds (all purchased from TargetMol, USA) or vehicle control once every two days for two weeks. Tumor volume was measured every two days. At the end of the treatment, tumors were harvested for sectioning and preparation of RNA or protein samples. All experimental procedures were approved by the Institutional Animal Care and Use Committee of FUSCC (approval number: FUSCC-IACUC-2024019), and conducted in compliance with the guidelines. The maximum tumor volume is no more than 2000 mm³.

### Tissue microarray (TMA), immunohistochemistry (IHC) and immunofluorescent staining (IF)

Clinical pancreatic tumor tissues obtained from surgical resections were fixed in 4% paraformaldehyde and embedded in paraffin before being sent to Wuhan Servicebio Technology for TMA fabrication and IHC staining. The pathological sections were dewaxed in xylene and rehydrated through a graded series of alcohols. Subsequently, the sections were treated with 3% hydrogen peroxide (H₂O₂) to quench endogenous peroxidase activity and subjected to antigen retrieval by boiling in sodium citrate buffer (pH 6.0). The sections were then incubated with 10% goat serum to block nonspecific binding sites, followed by incubation with primary antibodies and HRP- or fluorescence-conjugated secondary antibodies. The primary antibodies used for IHC included anti-NeuroD1 (1:200; 12081-1-AP, Proteintech), Ki67 (1:200; 27309-1-AP, Proteintech), CC3 (1:200; ab52101, Abcam), Cytokeratin 18 (1:200; 10830-1-AP, Proteintech), and NeuN (1:200; ab104224, Abcam). Hematoxylin and eosin (H&E) staining was performed for histological examination. Nuclei were counterstained with DAPI for 10 min. Pathological diagnosis and IHC scoring were performed by two experienced pathologists as described previously [26].

Cell coverslips were fixed in 4% paraformaldehyde (PFA) for 8 min and then permeabilized with PBS containing 0.2% Triton X-100 and 10% normal goat serum (NGS) for 30 min. For immunofluorescent staining, the primary antibodies used were anti-Map2 (1:200; 17490-1-AP, Proteintech) and anti-Tuj1 (1:200; 66375-1-Ig, Proteintech). The secondary antibodies and their dilutions were as follows: goat anti-mouse CoraLite594 (1:200; Proteintech, SA00013-3) and goat anti-rabbit CoraLite594 (1:200; Proteintech, SA00013-4). Finally, the coverslips were mounted using Fluoromount-G (Southern Biotechnology) and imaged under a Zeiss microscope.

### Western blotting (WB), co-immunoprecipitation (Co-IP) and mass spectrometry (MS)

Cell lysates or tumor tissues were prepared in RIPA buffer (Thermo Fisher Scientific) supplemented with protease and phosphatase inhibitor cocktails (Roche). The total protein concentration was determined using a BCA Protein Assay Kit (Beyotime). The protein supernatant was diluted in 5× loading buffer and heated at 100 °C for 10 min. Equal amounts of protein samples were separated by 10% SDS-PAGE and transferred onto PVDF membranes (Millipore). The membranes were blocked with 5% skim milk at room temperature for 1 h, followed by incubation with primary antibodies overnight at 4 °C. The primary antibodies used were as follows: anti-GAPDH (1:1000; 60004-1-Ig, Proteintech), anti-NeuroD1 (1:1000; 12081-1-AP, Proteintech), anti-Map2 (1:1000; 17490-1-AP, Proteintech), anti-Tuj1 (1:1000; 66375-1-Ig, Proteintech), anti-Neurog3 (1:1000; 100569-T08, SinoBiological), anti-MET (1:1000; 25869-1-AP, Proteintech), anti-Phospho-MET (1:1000; 30737-1-AP, Proteintech), anti-AKT (1:1000; 4060, CST), anti-Phospho-AKT (1:1000; 9271, CST), anti-PI3K (1:1000; 4257, CST), anti-Phospho-PI3K (1:1000; 17366, CST), anti-GFP (1:1000; 50430-2-AP, Proteintech), and anti-3xFlag (1:1000; 20543-1-AP, Proteintech). The following day, the membranes were washed three times with TBS buffer containing 0.05% Tween-20 for 10 min each. Subsequently, the membranes were incubated with HRP-conjugated secondary antibodies, including anti-mouse IgG (1:5000, CST, 7076) and anti-rabbit IgG (1:5000, CST, 7074), at room temperature for 2 h. Finally, protein band signals were detected using a Tanon 5200 chemiluminescence instrument.

The plasmid was transfected into HEK293 cells. After 48 h, the cells were lysed in NETN buffer (20 mM Tris-HCl, pH 8.0, 100 mM NaCl, 1 mM EDTA, 0.5% NP-40) containing Protease Inhibitor Cocktail (MCE, HY-K0010) for 10 min on ice. The lysates were then centrifuged at 12,500 rpm for 10 min. 10% of the supernatant was retained as the Input sample, while the remaining supernatant was equally divided and incubated with either 3 µg of IgG antibody or specific labeled antibody pre-bound to Protein A/G Agarose Resin (Yeasen, 36403ES08) for 4 h at 4 °C. The immunocomplexes were washed five times with NETN buffer and then sent to Shanghai Oebiotech for mass spectrometry analysis and data interpretation.

### Real-time quantitative PCR (qPCR)

Total RNA was extracted using RNAeasy™ Kit (R0027, Beyotime Biotechnology). cDNA was synthesized using HiScript III RT SuperMix for qPCR (R323-01, Vazyme, China).Q-PCR was performed in triplicate using SYBR qPCR Master Mix (Q711- 02, Vazyme, China) and the Bio-Rad iQ5 Multicolor Real-Time PCR Detection System. The differences in mRNA expression were calculated by the 2^−ΔΔCt^ method. Primer sequences are available as follows (all for human species):

GAPDH (forward:5’-GTCTCCTCTGACTTCAACAGCG-3’;reverse: 5’-ACCACCCTGTTGCTGTAGCCAA-3’); NeuroD1 (forward: 5’-GGTGCCTTGCTATTCTAAGACGC-3’;reverse: 5’-GCAAAGCGTCTGAACGAAGGAG-3’; Neurog3 (forward: 5’-CCTAAGAGCGAGTTGGCACTGA-3’;reverse: 5’-AGTGCCGAGTTGAGGTTGTGCA-3’); Map2(forward: 5’-AGGCTGTAGCAGTCCTGAAAGG-3’;reverse: 5’-CTTCCTCCACTGTGACAGTCTG-3’); Tuj1 (forward: 5’- TCAGCGTCTACTACAACGAGGC-3’;reverse: 5’- GCCTGAAGAGATGTCCAAAGGC-3’).

### Transwell assay

Transwell filters (24-well, 8 μm pore diameter, CLS3422, Corning, Corning, NY, USA) were placed in a 24-well plate, and the lower chamber was added with 400µL 10%FBS full medium. 1 × 104 cells were seeded in 200µL 10% FBS full medium in the Matrigel-free upper chamber. After 24 h of culture, the cells in the lower chamber were fixed with 4% paraformaldehyde, stained with 1% Crystal Violet and counted in randomly under microscope.

### Flow cytometry

For analyzing and purifying transdifferentiated pancreatic cancer cells, tumor cells were incubated with PE anti-human CD326 (EpCAM) Antibody (324206, Biolend) and APC anti-human CD56 (NCAM) Antibody (304610, Biolend) on ice for 30 min, diluted with 5 µl per million cells in 100 µl and washed 3 times on PBS. The analysis was performed using Beckman Coulter MoFlo Astrios EQ ultra-high-speed streaming sorting system.

### RNA sequencing

NeuroD1 and the vector were overexpressed in Panc-1 cells, and the stable cell line was obtained by puromycin screening. Total RNA was extracted according to RNAeasy™ Kit (R0027, Beyotime Biotechnology). Then, RNA sequencing was performed (Oebiotech, Shanghai China) and sequencing results were analyzed by using R-4.1.3 software.

### Computer simulation of potential Neuropathiazol targets

Based on the 2D structure of Neuropathiazol, its preliminary 3D structure was generated using the Builder module of the Molecular Operating Environment (MOE) software. Hydrogen atoms were added to the structure using the Wash function under the Amber10-EHT force field. The energy of the 3D structure was minimized with rigid solvent water, using an optimal gradient root mean square (RMS) cutoff of 0.1 kcal/mol/Å.

A pharmacophore represents the spatial arrangement of features necessary for molecular interaction with specific target receptors. PharmMapper extracts pharmacophore models from relevant databases and aligns query molecules with these models using a specific algorithm to predict potential targets [27–29]. Similar small molecule structures often exhibit similar biological activities. Based on this principle, SwissTargetPrediction extracted 2D/3D molecular fingerprints from a vast database of known active molecules. It then efficiently identifies similar molecules through fingerprint comparison, predicting potential targets for queried molecules [30]. GalaxySagittarius, developed by the Seok Research group at Seoul National University, is a reverse target-finding algorithm that integrates both small molecule similarity comparison and protein-small molecule spatial feature matching, enhanced by a machine learning algorithm (random forest) for improved matching accuracy [31]. The potential targets of Neuropathiazol were predicted using PharmMapper (Z-score >1), SwissTargetPrediction (Probability >0), and GalaxySagittarius (docking score < −23.5). The thresholds used were chosen based on commonly adopted criteria and tool recommendations. Specifically, (i) a Z-score >1 in PharmMapper has been suggested as indicative of reliable pharmacophore matching [29]; (ii) SwissTargetPrediction provides the top 100 putative targets ranked by probability, and previous studies commonly adopt this range as the cutoff for further analysis [30]; and (iii) in GalaxySagittarius, a docking score < − 23.5 has been used in prior virtual screening studies as indicative of favorable binding [31]. Individually, these methods identified 46, 45, and 93 probable targets, respectively. By combining and removing duplicates, these three methods collectively identified 175 likely targets for Neuropathiazol.

Simultaneously, we gathered known targets for pancreatic cancer from GeneCards v5.15 (https://www.genecards.org/), using “pancreatic cancer” as the search term. A total of 14,367 entries related to pancreatic cancer were collected, of which 11,061 proteins were associated with the disease. Applying a Score threshold of greater than 30, we screened out 336 targets with high correlation to pancreatic cancer. Through intersection analysis of the 175 targets obtained via database screening and the 336 targets identified through manual screening, we identified 15 targets that were recognized by both methods and confirmed by MOE software as capable of performing molecular docking with Neuropathiazol. The MOE software evaluates the interaction energy between proteins and Neuropathiazol, with the scoring value represented as the S value. A lower S value indicates a stronger affinity between the two entities.

### Molecular dynamics simulation

A 15 ns molecular dynamics (MD) simulation was performed to investigate whether the binding process between Neuropathiazol and the MET proteins could reach a stable state. Subsequently, the molecular mechanics generalized Born surface area (MM-GBSA) method was employed to accurately calculate the binding free energy between Neuropathiazol and the MET protein, thereby validating the previous prediction results. The Desmond program was used for this MD simulation. The prepared protein structure was solvated using the TIP3P explicit solvent model. The system’s shape was set as a cube, with the nearest distance between the solvent edge and the protein structure set to 10 Å. Chloride (Cl⁻) or sodium (Na⁺) ions were added to balance the system’s charge, setting the system’s net charge to 0. The salt concentration was set to 0.15 mol/L, establishing the protein-solvent system. The conformation of the complex was optimized to allow sufficient Brownian motion of solvent molecules and ions. This optimization process lasted 100 ps with a time step of 2 fs.

Following energy minimization, the system underwent a heating process using a gradual warming method. Initially, the Nose-Hoover thermostat was employed to raise the system’s temperature from 0 K to 300 K over a 1 ps relaxation period. Subsequently, the MTK (Martyna-Tobias-Klein) barostat was used to adjust the system to the appropriate density over another 1 ps, with isotropic coupling employed. Finally, the MD simulation was conducted at a constant temperature of 300 K and 1 atmosphere (atm) pressure for 15 ns, with a cutoff radius of 9 Å and a time step of 2.0 fs. The system output one frame every 10 ps for subsequent analysis.

Root mean square deviation (RMSD) statistical analysis of the MD simulation results indicated that the system began to converge after 2 ns, with the structure reaching equilibrium around 5 ns. The overall RMSD value fluctuated around 2.5 Å. For MM-GBSA binding free energy calculations, structure files from frames 500 to 1500 (corresponding to 5–15 ns) were selected, with calculations performed every 10 frames, resulting in a total of 100 sampled structures.

### Protein-protein docking

Starting with the gene name, we searched the amino acid sequence of the target protein from the UniProtKB database. The protein sequence obtained from UniProtKB was then input into the SWISS-MODEL platform for 3D structure prediction. SWISS-MODEL is an online homology modeling tool that generates high-quality protein structure models based on the input sequences and outputs them in PDB file format [32, 33]. For protein-protein docking, we employed GRAMM, a rigid docking tool that predicts protein binding patterns by randomly sampling multiple potential binding sites on the protein surface and calculating the interaction energy [34, 35]. Since GRAMM is a rigid docking tool and does not alter the shape of the proteins, it is well-suited for the initial exploration of possible binding sites between proteins.

### Single-dose pharmacokinetics and tissue distribution detection of Neuropathiazol in mice

Neuropathiazol (Compound No: T16288, TargetMol, USA) was dissolved in a solution consisting of 5% dimethyl sulfoxide (DMSO), 15% polyoxyethylene castor oil (HS-15), and 80% aqueous solution (containing 20% hydroxypropyl-β-cyclodextrin, HP-β-CD). The compound was administered to mice via intraperitoneal injection at a dose of 50 mg/kg and an administration volume of 10 mL/kg. Mouse plasma samples were collected at 0.083, 0.25, 0.5, 1, 2, 4, 6, 8 and 24 h post-administration. Plasma samples were prepared through protein precipitation with the addition of an internal standard, and the concentration of Neuropathiazol was quantified using high-performance liquid chromatography-tandem mass spectrometry (LC-MS/MS). The selection of detection time points was determined based on the plasma concentration profile of Neuropathiazol: the compound reached its peak concentration in plasma as early as 0.083 h after administration, followed by a gradual decline, and became barely detectable by 8 h post-administration. At the peak concentration time point (0.083 h) and two subsequent time points (0.25 h and 0.5 h), plasma samples and tissue samples (heart, liver, kidney, lung, and spleen) were collected from mice. Both plasma and tissue homogenates were prepared via protein precipitation with the addition of an internal standard, and the concentration of Neuropathiazol in these samples was quantified using LC-MS/MS. Pharmacokinetic parameters were calculated using the non-compartmental model with DAS 3.2.8 software [36–38]. All final data are presented as “mean ± standard deviation” (mean ± SD).

### Statistical analysis

All experiments were conducted at least in triplicate. Bioinformatics analysis was performed using R (version4.3.1) software. Data analysis was carried out using GraphPad Prism 9 software, and the results are presented as mean ± standard deviation (SD). For comparisons of continuous variables between two groups, Student’s t-test was used. Categorical variables were compared using chi-square tests or Fisher’s exact tests, depending on the data distribution. *P* < 0.05 was considered statistically significant.

### Online supplemental tables

Supplementary Table 1: RNA sequencing data of negative control Panc-1cells and NeuroD1-overexpressing Panc-1 cells. Supplementary Table 2: The promoter sequence of NeuroD1. Supplementary Table 3: Protein information that interacted with NeuroD1 obtained by the Co-IP experiment combined with mass spectrometry. Supplementary Table 4: Target Fishing List. Supplementary Table 5: Results of Protein-Protein docking between NeuroD1 and Neurog3. Supplementary Table 6: Detailed pharmacokinetic data, the in vivo dose-response curve, and tissue distribution data of Neuropathiazol.

## Results

### NeuroD1 is a protective factor in pancreatic cancer

**Fig. 1:**
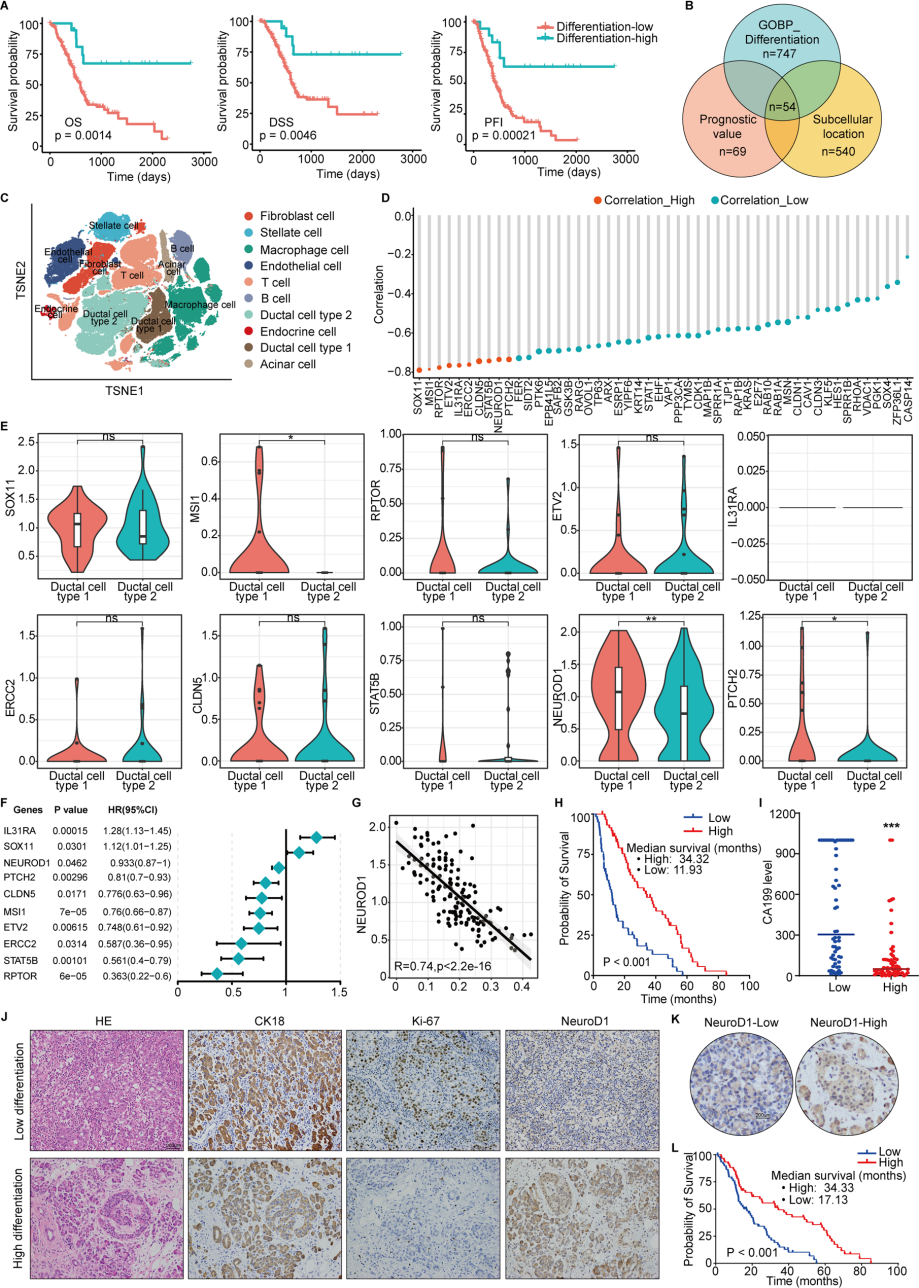
NeuroD1 is a protective factor in pancreatic cancer. **A** Patient samples in the TCGA (PAAD) database were categorized into two cohorts: one with high differentiation and another with low differentiation. Significant differences were observed between these cohorts in terms of overall survival (OS), disease-specific survival (DSS), and progression-free interval (PFI). **B** By incorporating the GOBP Negative Regulation of Epithelial Cell Differentiation gene set (747 genes), prognostic differential genes (COX and Kaplan-Meier analysis, 69 genes), along with subcellular localization (excluding secreted proteins while retaining membrane and intracellular proteins, 540 genes), intersection analysis was performed. 54 key genes were found to be involved in differentiation and malignant behavior of epithelial cells. **C** Publicly available single-cell data for pancreatic cancer (GSE154778, GSE155698, GSE111672, GSM4293555, and Peng et al. Cell Research 2019) were integrated, and detailed analyses of cell subpopulations were performed. **D** Correlation analysis between the gene expression levels and singlescore differentiation score of 54 key genes in ductal cells. **E** The violin plot displays the differential expression of the top 10 genes that are significantly correlated with the singlescore differentiation score in type I normal ductal cells and type II malignant ductal epithelial cells. Wilcox.test was used for the statistical analyses. Significance levels are denoted as follows: ns, not significant; *p < 0.05; **p < 0.01; ***p < 0.001. The exact *p*-values for each gene are as follows: SOX11 (0.99), MSI1 (0.019), RPTOR (0.39), ETV2 (0.76), ERCC2 (0.63), CLDN5 (0.49), STAT5B (0.13), NEUROD1 (*p* = 0.0075), PTCH2 (0.042). **F** HR (Hazard Ratio) analysis of the top 10 genes that are significantly associated with singlescore differentiation score in pancreatic cancer. **G** Correlation analysis between NeuroD1 and singlescore differentiation score in malignant ductal cells. **H** Survival analysis of 60 patients with highly differentiated pancreatic ductal adenocarcinoma (PDAC) and 60 patients with poorly differentiated PDACfrom FUSCC samples. **I** Preoperative CA199 levels in 60 patients with highly differentiated PDAC and 60 patients with poorly differentiated PDAC. **J** Representative images of H&E staining and IHC staining of CK18, Ki-67 and NeuroD1 proteins in the highly differentiated and poorly differentiated PDAC. **K** Representative images of high/low NeuroD1 protein expression in 120 PDAC samples from FUSCC. **L** Survival analysis of the NeuroD1 high expression cohort versus the NeuroD1 low expression cohort in 120 patients with PDAC from FUSCC. *P < 0.05, **P < 0.01, ***P < 0.001.NeuroD1 is a protective factor in pancreatic cancerNeuroD1 is a protective factor in pancreatic cancerNeuroD1 is a protective factor in pancreatic cancerNeuroD1 is a protective factor in pancreatic cancer

The gene set GOBP Negative Regulation of Epithelial Cell Differentiation includes a series of genes that exert negative regulatory influences on the differentiation of epithelial cells. These genes function by inhibiting or slowing the differentiation process, thereby maintaining the undifferentiated state of cells or facilitating alternative cell fate decisions. In our study, we initially utilized the TCGA database to compute singlescore values for each TCGA (PAAD) transcript-sequencing sample on the basis of the GOBP Negative Regulation of Epithelial Cell Differentiation gene set. This allowed us to predict the differentiation status of each sample and to classify it as belonging to a relatively highly differentiated group (differentiation-high) or a relatively poorly differentiated group (differentiation-low) (Fig. S1A). An analysis of patient prognostic data revealed that overall survival (OS), disease-specific survival (DSS), and progression-free interval (PFI) were significantly greater in the relatively highly differentiated group than in the relatively poorly differentiated group (Fig. 1A). We conducted an intersection analysis that incorporated the GOBP Negative Regulation of Epithelial Cell Differentiation gene set (747 genes), prognostic differential genes (COX and Kaplan‒Meier analysis, 69 genes), and genes encoding proteins with specific subcellular localizations (excluding secreted proteins but including membrane and intracellular proteins, 540 genes). Through this process, we identified 54 key genes that may be involved in epithelial cell differentiation and malignant behavior (Fig. 1B). We subsequently integrated and analyzed publicly available single-cell data on pancreatic cancer from the GSE154778, GSE155698, GSE111672, GSM4293555 datasets and from Peng et al. *Cell Research 2019* [39]. Following Peng et al.’s annotation method for ductal cells, we classified ductal cells as type I (normal) ductal cells or type II (malignant) ductal epithelial cells (Fig. 1C). Analysis of the correlation between gene expression levels and singlescore differentiation scores associated with these 54 key genes in ductal cells revealed a significant association (Fig. 1D). The violin plot shown in Fig. 1E displays the differential expression of the top 10 genes whose expression is significantly correlated with the singlescore differentiation score in type I normal ductal cells and type II malignant ductal epithelial cells. We observed that decreased NeuroD1 expression was most pronounced in type II malignant ductal epithelial cells. Further hazard ratio (HR) analysis indicated that NeuroD1 serves as a protective factor in pancreatic cancer (Fig. 1F, HR < 1, *P* = 0.05). In malignant ductal cells, NeuroD1 expression correlated negatively with the singlescore differentiation score (Fig. 1G, *R* = −0.74). Our analyses of data from public databases preliminarily suggest that high expression of NeuroD1 is associated with a highly differentiated phenotype in pancreatic cancer and that NeuroD1 may function as a protective factor in pancreatic cancer.

Next, we conducted a survival analysis of 60 patients with highly differentiated pancreatic ductal adenocarcinoma and 60 patients with poorly differentiated pancreatic ductal adenocarcinoma who were treated at our center. Our findings revealed that patients with highly differentiated pancreatic cancer had significantly longer survival times than did those with poorly differentiated pancreatic cancer (Fig. 1H, median survival: 34.32 months vs. 11.93 months, *P* < 0.001). Additionally, the preoperative CA199 levels were notably lower in the highly differentiated group (Fig. 1I). These results suggest that highly differentiated pancreatic cancers indeed exhibit a lower degree of malignancy and are associated with improved patient outcomes. To further investigate this phenomenon, we used IHC staining to measure the levels of expression of CK18 (cytokeratin 18, a marker of epithelial cell differentiation [40]), Ki-67 (a marker of cell proliferation [41]), and NeuroD1 in highly differentiated and poorly differentiated pancreatic ductal adenocarcinomas. The results indicated that highly differentiated pancreatic cancers presented reduced cell proliferation and increased NeuroD1 protein expression (Fig. 1J). The findings suggest that elevated NeuroD1 expression is correlated with increased differentiation of pancreatic cancer cells. We then quantified the expression of NeuroD1 protein in another 120 pancreatic ductal adenocarcinomas present in patients treated at our center, categorizing them into high and low NeuroD1 expression groups (Figs. 1K and S1B). Analysis of the correlation of NeuroD1 expression with patient survival time revealed that patients with high NeuroD1 expression had significantly longer survival times (Fig. 1L). In conjunction with the results of bioinformatics analyses of data obtained from public databases and of our analyses of patient tissue samples, we can conclude that NeuroD1 expression is associated with increased levels of cell differentiation and improved patient outcomes in patients with pancreatic cancer. These findings suggest that NeuroD1 may serve as a protective factor in pancreatic cancer.

### NeuroD1 inhibits proliferation of pancreatic cancer cells

**Fig. 2:**
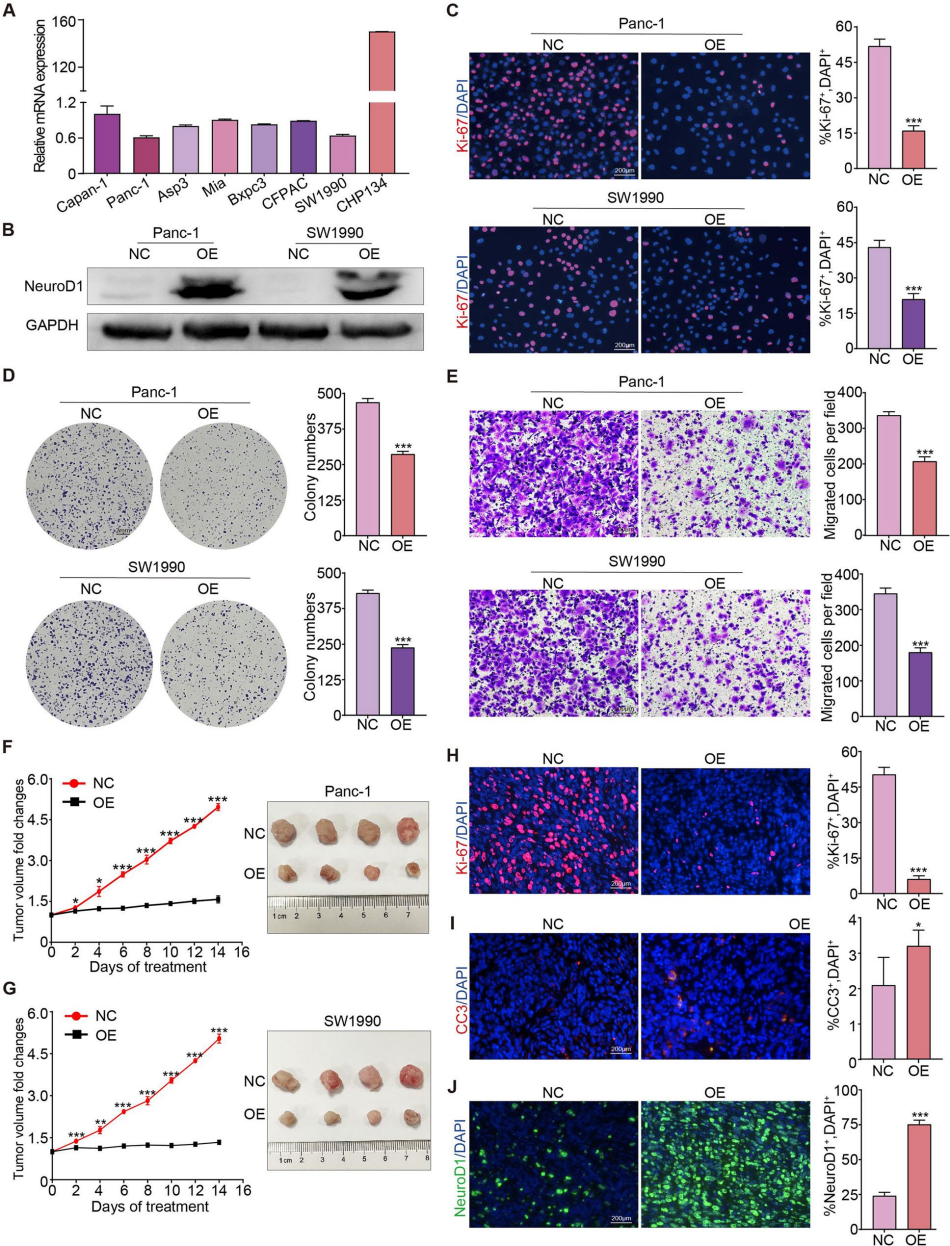
NeuroD1 inhibits proliferation of pancreatic cancer cells. **A** The expression level of NeuroD1 in common pancreatic cancer cell lines and human neuroblastoma cell line CHP134 was detected by RT-qPCR. **B-E** NeuroD1-overexpressing stable cell lines (OE) in Panc-1 and SW1990 cells were constructed by lentiviral infection, using the same cell line transfected with empty plasmid as a negative control (NC). **B** Western Blotting verified the successful construction of OE cell lines. **C** IF staining was used to stain Ki-67 protein (in red color) in OE and NC cells to label proliferating cells. Using DAPI to counterstain the cell nucleus (in blue color). The percentage of Ki-67^+^ cells was quantified. Scale bar: 200 μm. **D** The proliferation ability of OE and NC pancreatic cancer cells was detected by clonal formation experiment, and the number of cell colonies was counted. **E** The migration ability of OE and NC pancreatic cancer cells was detected by Transwell experiment, and the number of migrated cells per field was counted. Scale bar: 200 μm. **F-J** Subcutaneous pancreatic cancer model of Panc-1/SW1990 cells was constructed to explore NeuroD1 functions in vivo. **F** Monitor the volume change of subcutaneous tumors (Panc-1 cells) every 2 days and displayed by line charts. On the 14th day of subcutaneous tumor construction, the tumors were separated and photographed to show the tumor size. **G** Monitor the volume change of subcutaneous tumors (SW1990 cells) every 2 days and displayed by line charts. On the 14th day of subcutaneous tumor construction, the tumors were separated and photographed to show the tumor size. **H-J** Frozen sections were made from subcutaneous tumor tissue (Panc-1 cells). IF staining of Ki-67 (in red color, (**H**)) and CC3 protein (in red color, (**I**)) was performed to show proliferation and apoptosis; of NeuroD1 protein (in green color, (**J**)) was performed to detect its expression levels. Using DAPI to counterstain the cell nuclei (in blue color). Scale bar: 200 μm. The percentage of Ki-67^+^, CC3^+^, and NeuroD1^+^ cells per field were quantified. *P < 0.05, **P < 0.01, ***P < 0.001

Next, we explored the specific role of NeuroD1 in pancreatic cancer. As shown in Fig. 2A, we assessed the expression levels of NeuroD1 in common pancreatic cancer cell lines and in the human neuroblastoma cell line CHP134 via RT-qPCR. Among the pancreatic cancer cell lines, Panc-1 and SW1990 cells exhibited the lowest levels of NeuroD1 expression; using these cell lines, we established stable NeuroD1-overexpressing (OE) cell lines via lentiviral infection. The same cell lines transfected with an empty plasmid served as negative controls (NCs). Western Blotting confirmed the successful establishment of the NeuroD1-OE cell lines (Fig. 2B).

Through IF staining for the Ki-67 protein, which labels proliferating cells, and colony formation assays, we observed that NeuroD1 overexpression significantly decreased the proliferative capacity of pancreatic cancer cells (Fig. 2C, D). Transwell assays revealed that NeuroD1 also decreased the migratory ability of pancreatic cancer cells (Fig. 2E). We established subcutaneous tumor models by inoculating nude mice with Panc-1 and SW1990 cells and found that NeuroD1 overexpression hindered the progression of pancreatic cancer in vivo (Fig. 2, F and G). Subcutaneous tumor tissues were harvested and subjected to IF staining for the Ki-67 and Cleaved Caspase-3 (CC3) proteins [42, 43]. Our findings revealed a marked reduction in proliferative potential (Fig. 2H) and an increase in apoptosis (Fig. 2I) in the pancreatic cancer cells. IF staining for the NeuroD1 protein confirmed the successful establishment of the NeuroD1 overexpression model (Fig. 2J). Collectively, these results suggest that upregulation of NeuroD1 expression suppresses the proliferation of pancreatic cancer cells both in vitro and in vivo.

### NeuroD1 induces pancreatic cancer cells to transdifferentiate into neurons

**Fig. 3:**
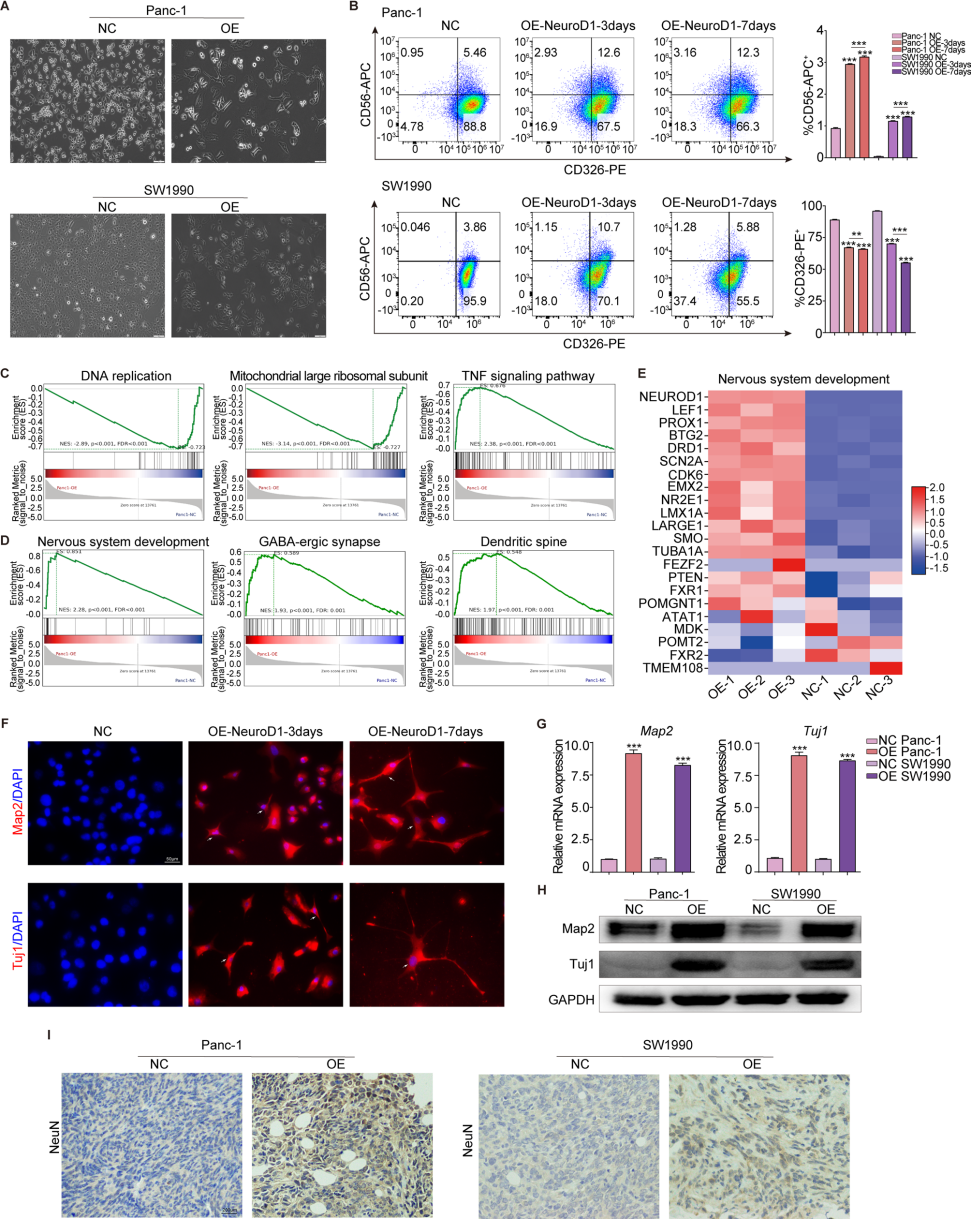
NeuroD1 induces pancreatic cancer cells to transdifferentiate into neurons. **A** Overexpression of NeuroD1 induces the morphological alteration of pancreatic cancer cells into neuron-like cell morphology. Scale bar: 100 μm. **B** Flow cytometry antibodies of CD56-APC (a neuron marker) and CD326-PE (an epithelial cell marker) were used to stain pancreatic cancer cells at day 0, 3, and 7 after NeuroD1 overexpression, and subsequent flow cytometry was performed. The percentage of CD56^+^ and CD326^+^ cells in each group was analyzed. **C-E** Negative control cells and NeuroD1 overexpressed Panc-1 cells were collected for transcriptome sequencing and subsequent analysis. **C** GSEA enrichment analysis of the differentially expressed genes showed that the pathways related to DNA replication and mitochondrial function were significantly down-regulated in NeuroD1-overexpressing cells, while the TNF signaling pathway was significantly up-regulated. **D** GSEA enrichment analysis revealed extensive enrichment of differentially expressed genes in many neuron-related pathways. **E** Heat map of the pathways of Nervous system development. **F** At days 3 and 7 following NeuroD1 overexpression, CD56^+^ cells were subjected to IF staining for Map2 and Tuj1 proteins (in red color) to examine the morphology of the cytoskeleton. Cells at day 0 post-NeuroD1 overexpression served as negative controls. Using DAPI to counterstain the cell nuclei (in blue color). Scale bar: 50 μm. **G** RT-qPCR was used to detect the expression levels of Map2 and Tuj1 in negative control (NC) and NeuroD1 overexpressed (OE) pancreatic cancer cells. **H** Western Blotting was used to detect the expression levels of Map2 and Tuj1 in negative control (NC) and NeuroD1 overexpressed (OE) pancreatic cancer cells. **I** Representative images of the IHC staining for NeuN protein on subcutaneous tumor tissues from mice in previous in vivo experiments. Scale bar: 200 μm

NeuroD1-overexpressing pancreatic cancer cells appeared to undergo morphological alterations and to exhibit a neuron-like cell morphology (Fig. 3A). To evaluate the cellular changes induced by NeuroD1 overexpression, we performed flow cytometry using antibodies specific for CD56 (a neuronal marker) and CD326 (an epithelial cell marker) on days 0, 3, and 7 after inducing NeuroD1 overexpression [44–46]. Our results indicated that as NeuroD1 overexpression increased, the percentage of CD326-positive cells steadily decreased, and the percentage of CD56-positive cells steadily increased. Concurrently, the proportion of cells coexpressing CD326 and CD56 also increased, suggesting progressive transdifferentiation of cancer cells into neurons (Fig. 3B). To explore the mechanisms underlying this transformation, we performed RNA sequencing analysis of negative control Panc-1 cells (NC) and NeuroD1-overexpressing Panc-1 cells (OE). The detailed RNA sequencing data are shown in Table S1. As shown in Fig. 3C, GSEA enrichment analysis of the differentially expressed genes (DEGs) between OE and NC cells showed that GSEA pathways associated with DNA replication and mitochondrial function were significantly downregulated in NeuroD1-overexpressing cells, indicating that biological processes associated with promoting cell proliferation and growth were inhibited. Conversely, the activity of the TNF signaling pathway was significantly increased, suggesting that cell biological processes that are negatively associated with proliferation and growth, such as apoptosis and necrosis, are promoted in NeuroD1-overexpressing cells. This finding is consistent with our previous observations that overexpression of NeuroD1 suppresses the proliferation of pancreatic cancer cells. Another significant discovery was that the identified DEGs were highly enriched in neuron-related pathways, such as nervous system development, GABAergic synapses, and dendritic spines (Fig. 3D). These findings suggest that NeuroD1 overexpression may induce the transdifferentiation of pancreatic cancer cells into neurons. We constructed a heatmap of neural developmental pathways and identified NeuroD1 as a pivotal gene within this pathway. The heatmap also confirmed that NeuroD1-overexpressing cells had been successfully generated (Fig. 3E).

Microtubule-associated protein 2 (Map2), class III β-tubulin (Tuj1), and neuronal nuclei (NeuN) protein are important protein markers in neurons [47–49]. Map2 and Tuj1 are key neuronal cytoskeletal proteins; Tuj1 is frequently utilized as a marker of early neuronal differentiation, and NeuN is commonly employed as a marker of mature, differentiated neurons. We sorted the CD56-positive NeuroD1-overexpressing cells and subjected them to IF staining to visualize their cytoskeletal morphology by staining for Map2 and Tuj1. As shown in Fig. 3F, in contrast to control cells, some NeuroD1-overexpressing cells exhibited neuron-like morphology by the 3rd day after NeuroD1 overexpression; by the 7th day, these cells displayed obvious neuronal morphology. These findings support the idea that NeuroD1 indeed induces the transdifferentiation of pancreatic cancer cells into neurons and indicate that the degree of transdifferentiation increases over time with sustained NeuroD1 overexpression. Furthermore, through RT-qPCR (Fig. 3G) and Western Blotting (Fig. 3H) experiments, we confirmed that the expression of Map2 and Tuj1 is upregulated in NeuroD1-overexpressing pancreatic cancer cells at both the gene and protein levels. Concurrently, IHC staining for NeuN was conducted on mouse subcutaneous tumor tissues collected from prior in vivo experiments. While control tissues contained virtually no NeuN-positive cells, a significant proportion of NeuN-positive cells were found among the NeuroD1-overexpressing cells, suggesting that some of the pancreatic cancer cells had transdifferentiated into mature neuron-like cells (Fig. 3I). Additionally, we sorted two distinct cell populations, one positive for CD56 and the other for CD326, by flow cytometry and used these sorted cell populations to establish subcutaneous xenograft tumors in mice. Only the CD326-positive cells gave rise to tumors; the CD56-positive group failed to form tumors (data not shown). Collectively, these results imply that NeuroD1 facilitates the transdifferentiation of pancreatic cancer cells into neuron-like cells.

### Neuropathiazol upregulates NeuroD1 expression in pancreatic cancer

NeuroD1 can induce the transformation of pancreatic cancer cells from a malignant phenotype to neurons with a benign phenotype; thus, screening for drugs that regulate and induce NeuroD1 expression may facilitate the treatment of pancreatic cancer. Our previous research has demonstrated that the expression of NeuroD1 in medulloblastoma could be regulated by epigenetic mechanisms [22]. However, H3K27 methylation inhibitors, which can used to modulate such epigenetic changes, are often associated with significant toxicity and side effects. Given these limitations, it is imperative to identify more efficient and less toxic drugs that can effectively regulate NeuroD1 expression specifically in pancreatic cancer. To achieve this goal, we conducted a comprehensive drug screening experiment. In parallel, we used the EZH2 inhibitor UNC1999 as a control to evaluate its impact on NeuroD1 expression in pancreatic cancer cells (Fig. S2A-D). Our findings revealed that UNC1999 did not affect the expression levels of NeuroD1 in pancreatic cancer.

**Fig. 4:**
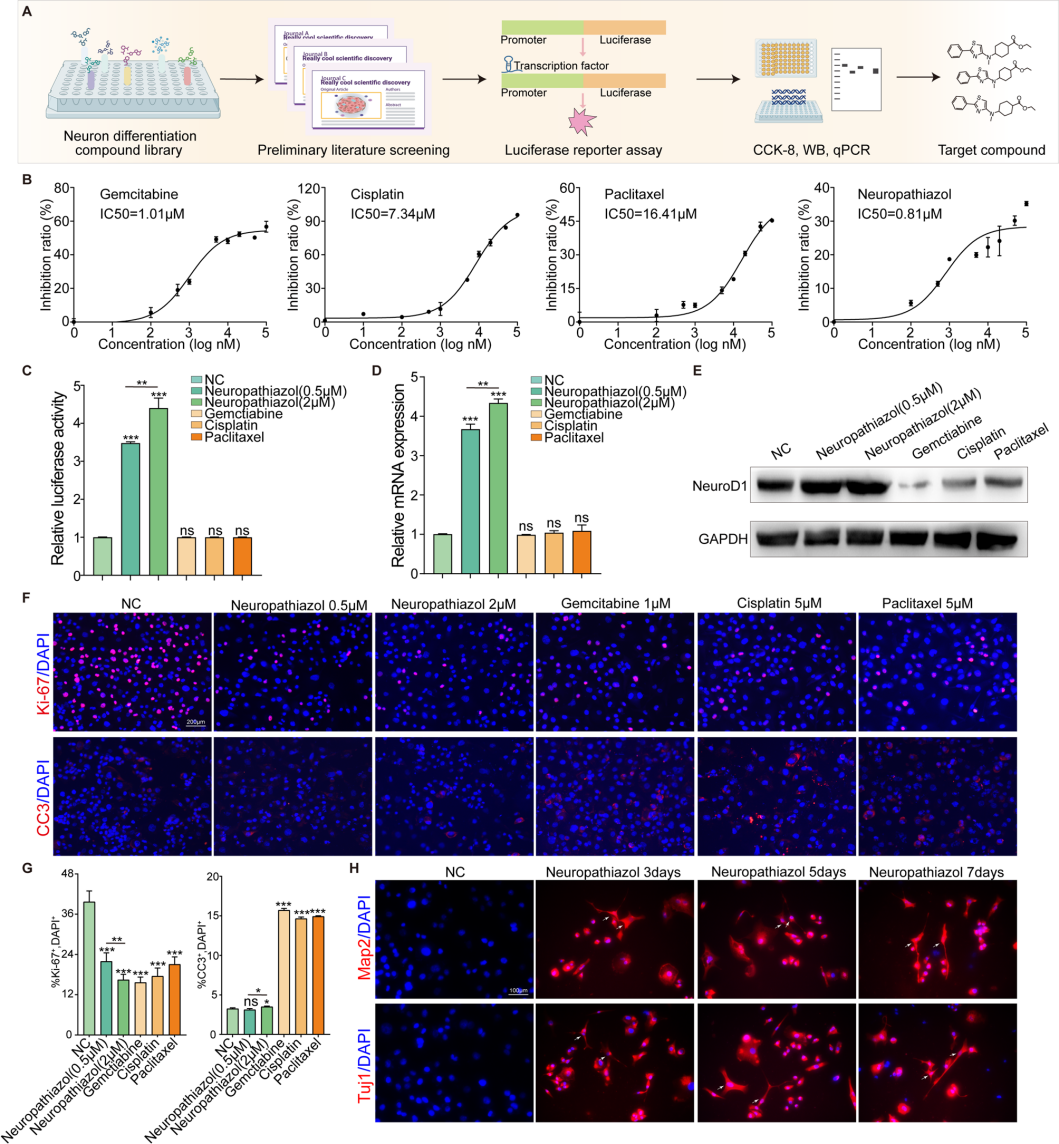
Neuropathiazol upregulates NeuroD1 expression in pancreatic cancer. **A** A schematic diagram illustrates the process for screening drugs that induce and regulate NeuroD1 expression. Initially, from a library of 310 compounds associated with neuronal differentiation, 60 compounds that potentially induce NeuroD1 expression were preliminarily selected based on literature evidence. Subsequently, a NeuroD1 promoter luciferase assay identified 13 compounds that regulate NeuroD1 gene expression. Finally, the effects of these compounds on cell proliferation were assessed using the CCK-8 assay, while NeuroD1 expression was confirmed through qPCR and Western Blotting. Neuropathiazol was identified as a small molecule capable of inducing NeuroD1 upregulation with low cytotoxicity. **B** The CCK-8 assay was employed to detect the half-maximal inhibitory concentrations (IC50) of Neuropathiazol, Gemcitabine, Cisplatin, and Paclitaxel on Panc-1 cells. **C-G** Panc-1 cells were treated with PBS, Neuropathiazol (0.5 µM), Neuropathiazol (2 µM), Gemcitabine (1 µM), Cisplatin (5 µM), and Paclitaxel (5 µM), respectively, for 48 h. Then, the following experiments were conducted. **C** NeuroD1 promoter luciferase assay was used to evaluated NeuroD1 promoter activity. **D** RT-qPCR was used to detect the expression level of NeuroD1. **E** Western Blotting was used to detect the expression level of NeuroD1. **F** IF staining of Ki-67 and CC3 protein (in red color) was performed to assess cell proliferation and apoptosis. Nuclei were counterstained with DAPI (in blue color). Scale bar: 200 μm. **G** The percentage of Ki-67^+^ and CC3^+^ cells were quantified. *P < 0.05, **P < 0.01, ***P < 0.001. **H** Panc-1 cells were treated with Neuropathiazol (2 µM). At days 0, 3, and 7 post-treatment, IF staining of Map2 and Tuj1 proteins (in red color) was performed to visualize the cytoskeletal morphology. Nuclei were counterstained with DAPI (in blue color). Scale bar: 100 μm

First, we initially identified 60 compounds that may induce NeuroD1 expression on the basis of evidence from a library of 310 compounds related to neuronal differentiation. Using a NeuroD1 promoter luciferase assay, we subsequently identified 17 compounds that regulate the expression of the NeuroD1 gene. Finally, the effects of these compounds on cell proliferation were evaluated via the CCK-8 assay, and the expression of NeuroD1 was verified via qPCR and Western Blotting. We identified Neuropathiazol as a small-molecule compound that is capable of inducing the upregulation of NeuroD1 expression with low cytotoxicity. Figure 4A shows a schematic diagram of the drug screening process, Table S2 provides the promoter sequence of NeuroD1, and Fig. S2 shows the results of NeuroD1 promoter luciferase assays (Fig. S2E, F), CCK-8 assays (Fig. S2I, J) and qPCR assays (Fig. S2G, H) for Panc-1 and SW1990 cells following treatment with various compounds in the neuronal differentiation compound library.

We used the CCK-8 assay to determine the half-maximal inhibitory concentrations (IC50s) of Neuropathiazol and commonly used first-line chemotherapeutic agents for pancreatic cancer, including gemcitabine, cisplatin, and paclitaxel. Our findings revealed that Neuropathiazol is highly effective in inhibiting the proliferation of pancreatic cancer cells, demonstrating even superior anti-tumor activity compared to conventional first-line chemotherapy drugs (Fig. 4B). Moreover, within a specific concentration range, Neuropathiazol does not impair the proliferative capacity of normal pancreatic epithelial cells (Fig. S2K). Treatment of Panc-1 cells with PBS, 0.5 µM Neuropathiazol, 2 µM Neuropathiazol, 1 µM gemcitabine, 5 µM cisplatin, or 5 µM paclitaxel for 48 h and subsequent measurement of relative luciferase activity to assess NeuroD1 promoter activity showed that the first-line chemotherapeutic drugs commonly used to treat pancreatic cancer did not affect NeuroD1 promoter activity in Panc-1 cells. In contrast, treatment with Neuropathiazol resulted in a significant and concentration-dependent increase in NeuroD1 promoter activity (Fig. 4C). We also examined changes in the expression of NeuroD1 in Panc-1 cells at the gene and protein levels following treatment with Neuropathiazol and various chemotherapeutic drugs via qPCR and Western blotting. While gemcitabine, cisplatin and paclitaxel induced no changes in NeuroD1 expression, treatment with Neuropathiazol caused a concentration-dependent upregulation of NeuroD1 expression in Panc-1 cells (Fig. 4D, E). These results suggest that Neuropathiazol induces upregulation of NeuroD1 expression in pancreatic cancer cells and that NeuroD1 exhibits potent antitumor activity in vitro.

To further explore the antitumor activity of Neuropathiazol, we treated Panc-1 cells with various drugs at the abovementioned concentrations for 48 h and performed IF staining for Ki-67 and CC3 to allow observation of cell proliferation and apoptosis (Fig. 4F, G). IF staining for the Ki-67 protein revealed that, compared with the control, treatment with either Neuropathiazol or chemotherapeutic drugs inhibited cell proliferation. In addition, the higher the concentration of Neuropathiazol used, the more obvious the inhibition of proliferation. IF staining for the CC3 protein revealed that, compared with the control, treatment with the chemotherapeutic agents significantly increased apoptosis, whereas Neuropathiazol caused only a slight increase or no increase in apoptosis. Thus, it can be concluded that Neuropathiazol has effective antitumor activity in vitro. Next, we performed IF staining for Map2 and Tuj1 at various time points after the initiation of Neuropathiazol treatment to permit observation of the morphology of the cytoskeleton. As shown in Fig. 4H, treatment with Neuropathiazol induced the morphological transformation of the pancreatic cancer cells into cells that displayed a neuronal phenotype. As the treatment progressed, these cells progressively evolved into cells with a morphology similar to that of mature neurons. On the basis of the above findings, Neuropathiazol was identified as a drug that can induce and regulate the expression of NeuroD1. Neuropathiazol upregulates NeuroD1 expression in pancreatic cancer cells and induces the transdifferentiation of pancreatic cancer cells into neurons.

### Neuropathiazol inhibits the growth of pancreatic cancer in vivo

**Fig. 5:**
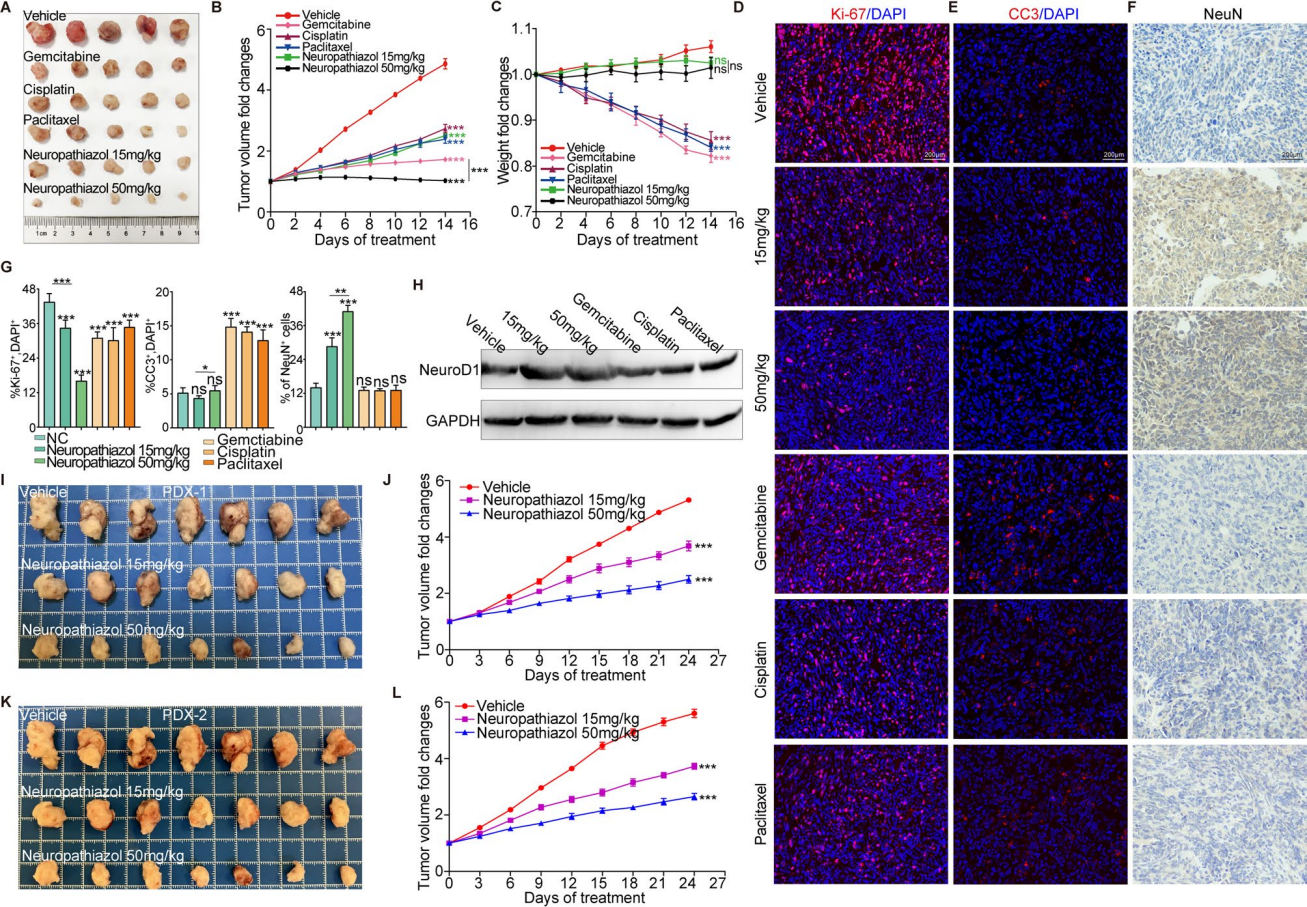
Neuropathiazol inhibits the growth of pancreatic cancer in vivo. **A-H** mouse subcutaneous tumor model was established using Panc-1 cells. When the tumor volume reached 200 mm³, the mice were randomly assigned into six groups. These groups received the following treatments, respectively: Neuropathiazol (15 mg/kg or 50 mg/kg), gemcitabine (20 mg/kg), cisplatin (10 mg/kg), and paclitaxel (15 mg/kg); a control group was administered vehicle. The drugs were delivered via intraperitoneal injection, with treatments administered every two days over a two-week period. **A** After two weeks of drug treatment, subcutaneous tumors were collected and photographed. **B** The volume change of subcutaneous tumors was monitored every 2 days, as shown in line charts. **C** The changes in body weight of the mice were monitored every 2 days and displayed by line charts. **D** Subcutaneous tumor tissues were harvested and IF staining for Ki-67 protein (in red color) was performed to label proliferating cells. Nuclei were counterstained with DAPI (in blue color). Scale bar: 200 μm. **E** IF staining for CC3 protein (in red color) was performed to label apoptotic cells. Nuclei were counterstained with DAPI (in blue color). Scale bar: 200 μm. **F** IHC staining for NeuN protein was performed to label pancreatic cancer cells that have transdifferentiated into mature neurons. Scale: 200 μm. **G** The percentage of Ki-67^+^ cells, CC3^+^ cells and NeuN^+^ cells were quantified. * *p* < 0.05, ** *p* < 0.01, *** *p* < 0.001. **H** Western Blotting was used to detect the expression levels of NeuroD1. **I-L** PDAC tissues were collected from two pancreatic cancer patients and implanted subcutaneously into NSG mice to establish patient-derived xenograft (PDX) models, designated as PDX-1 and PDX-2. When the tumor volume reached approximately 200 mm³, the mice were randomly assigned into three groups and treated with vehicle, Neuropathiazol (15 mg/kg), and Neuropathiazol (50 mg/kg), respectively. The treatment was administered via intraperitoneal injection, with a frequency of once every three days. **I** In the PDX-1 model, mouse tumors were harvested and photographed on day 24 after administration. **J** In the PDX-1 model, changes in the tumor volumes were monitored every 3 days, as shown in line charts. **K** In the PDX-2 model, mouse tumors were harvested and photographed on day 24 after administration. **L** In the PDX-2 model, changes in the tumor volumes were monitored every 3 days, as shown in line charts

Next, we conducted a series of in vivo experiments in which we further explored the antitumor function of Neuropathiazol in vivo. Panc-1 cells were used to construct a mouse subcutaneous tumor model. When the volumes of the tumors reached nearly 200 mm³, the mice were randomly divided into six groups and treated with Neuropathiazol (15 mg/kg or 50 mg/kg), gemcitabine (20 mg/kg), cisplatin (10 mg/kg), or paclitaxel (15 mg/kg). The control group was treated with vehicle. The compounds and drugs were administered by intraperitoneal injection once every two days for a period of two weeks. At the end of the two-week period, the subcutaneous tumors were collected and photographed. As shown in Fig. 5A and B, Neuropathiazol, as well as the three chemotherapeutic drugs that we tested, inhibited the growth of subcutaneous tumors in vivo. The degree to which tumor growth was inhibited after administration of Neuropathiazol at 15 mg/kg was similar to that after administration of the chemotherapeutic drugs, whereas treatment with Neuropathiazol at 50 mg/kg more significantly inhibited tumor growth. We monitored the weight of the animals during the period of drug administration and found that whereas the chemotherapeutic drugs caused significant weight loss, Neuropathiazol treatment did not result in significant changes in weight (Fig. 5C). These findings suggest that treatment with Neuropathiazol does not cause weight loss as a side effect. Subcutaneous tumors were collected and subjected to IF staining for the Ki-67 and CC3 proteins. The results indicated that both Neuropathiazol and the chemotherapeutic drugs significantly inhibited the proliferation of pancreatic cancer cells in vivo and that treatment with higher concentrations of Neuropathiazol yielded more pronounced antiproliferative effects (Fig. 5D and G). Moreover, while the chemotherapeutic drugs promoted apoptosis in pancreatic cancer cells, Neuropathiazol did not significantly increase apoptosis in the short term (Fig. 5E, G). IHC staining for the NeuN protein in subcutaneous tumors revealed a significant increase in the proportion of NeuN-positive cells following Neuropathiazol treatment, and this increase was concentration-dependent (Fig. 5F and G). And the neuroD1 expression in subcutaneous tumors was examined by Western blotting. Our results showed that Neuropathiazol upregulated NeuroD1 expression in the tumor tissues, in contrast to the chemotherapeutic drugs, which had no effect (Fig. 5H).These findings suggest that Neuropathiazol induces the transdifferentiation of pancreatic cancer cells into neurons in a concentration-dependent manner. In contrast, the tested chemotherapeutic drugs did not induce corresponding transdifferentiation of the cells.

We subsequently collected pancreatic ductal adenocarcinoma tissues from two pancreatic cancer patients and implanted them subcutaneously into NSG mice to construct patient-derived xenograft (PDX) models. When the tumor volume reached approximately 200 mm³, the mice in which the tumor tissue had been implanted were randomly divided into three groups. The groups of mice were treated either with vehicle, with 15 mg/kg Neuropathiazol, or with 50 mg/kg Neuropathiazol. The drugs were administered by intraperitoneal injection once every three days, and the changes in tumor volume were monitored during the treatment. On day 24 after drug treatment, the tumors were collected and photographed. As shown in Fig. 5I-5F, Neuropathiazol significantly inhibited the progression of patient-derived subcutaneous tumors in mice, and its inhibitory effect became more pronounced as the concentration of Neuropathiazol increased. These in vivo experiments clearly show that Neuropathiazol effectively inhibits the progression of pancreatic cancer in vivo.

Building on this foundation, we further investigated the therapeutic efficacy of Neuropathiazol in combination with chemotherapeutic agents. A subcutaneous tumor model in mice was established using Panc-1 cells. When the volumes of the tumors reached nearly 200 mm³, the mice were randomly divided into four groups and treated with the control vehicle, gemcitabine (20 mg/kg), Neuropathiazol (50 mg/kg), and Neuropathiazol (50 mg/kg) combined with gemcitabine (20 mg/kg), respectively. All treatments were administered via intraperitoneal injection at a frequency of once every two days, with the treatment duration lasting for two weeks. After two weeks, the subcutaneous tumors of the mice were collected and photographed. As shown in Fig. S3A and S3B, Neuropathiazol (50 mg/kg) exhibited a more significant ability to inhibit in vivo tumor growth compared to gemcitabine (20 mg/kg); notably, the combination of Neuropathiazol (50 mg/kg) and gemcitabine (20 mg/kg) further enhanced the inhibitory effect on subcutaneous tumor growth. IF staining of the Ki-67 protein in subcutaneous tumors also revealed that the combination of Neuropathiazol and gemcitabine further suppressed the in vivo proliferation of pancreatic cancer cells (Fig. S3C and S3D). These findings suggest that the combination of the transdifferentiating agent Neuropathiazol with chemotherapeutic drugs may exert a more prominent tumor-suppressive effect by further eliminating residual tumor cells.

### Neuropathiazol binds to the MET protein and inhibits its phosphorylation

Next, we aimed to elucidate the targets of Neuropathiazol and the specific mechanisms that underlie the Neuropathiazol-induced upregulation of NeuroD1 expression.

**Fig. 6:**
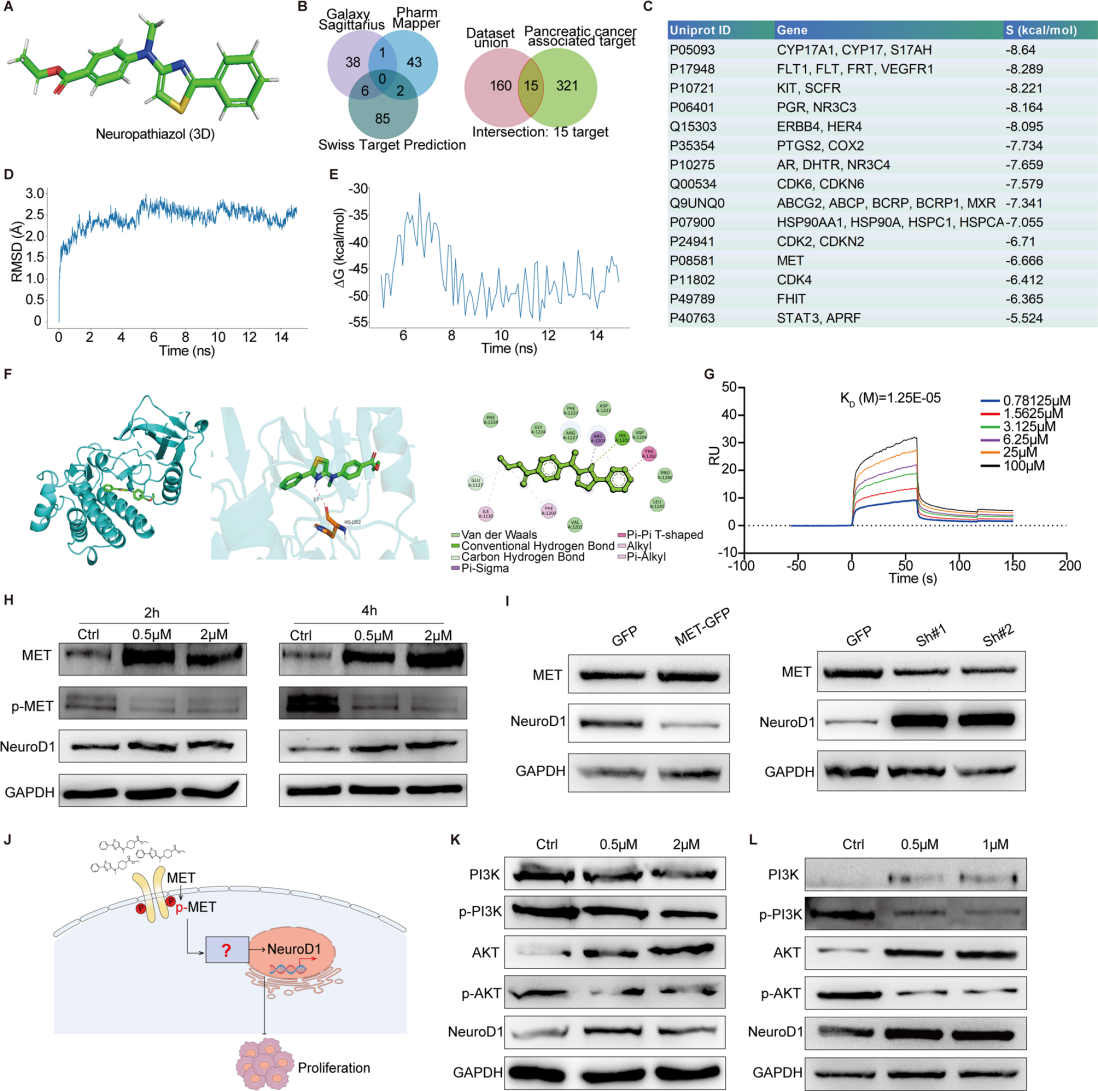
Neuropathiazol binds to the MET protein and inhibits its phosphorylation. **A** Preliminary 3D structure of Neuropathiazol generated by Molecular Operating Environment (MOE) software. **B** PharmMapper (with a Z-score > 1), SwissTargetPrediction (with a Probability > 0), and the GalaxySagittarius method (with a docking score < -23.5) was used to predict the potential targets of Neuropathiazol. These methods identified a total of 175 potential targets for Neuropathiazol. Simultaneously, known targets for pancreatic cancer were gathered from GeneCards v5.15 (https://www.genecards.org/), using “pancreatic cancer” as the search term. Applying a Score threshold of greater than 30, a total of 336 potential targets for Neuropathiazol were identified. Through intersection analysis of the 175 targets obtained via database screening and the 336 targets identified through manual screening, 15 targets that were recognized and confirmed by MOE software as capable of performing molecular docking with Neuropathiazol. **C** The 15 potential targets of Neuropathiazol were identified through both algorithmic and manual screening methods. The UniProt ID serves as a unique identifier for proteins within the UniProt database. The S value is utilized to evaluate the strength of the association between the compound and its predicted target; a smaller S value indicates a stronger association. **D** Root Mean Square Deviation (RMSD) of the interaction between Neuropathiazol and the MET protein (0–15 ns). **E** The MM-GBSA binding free energy between Neuropathiazol and the MET protein (5-15 ns). **F** The conformation of the complex formed between Neuropathiazol and the MET protein. Neuropathiazol binds to MET protein via a hydrogen bond with His-1202. **G** Surface plasmon resonance (SPR) technology was employed to determine the affinity between MET protein and Neuropathiazol. **H** Panc-1 cells were treated with PBS or Neuropathiazol (0.5 µM or 2 µM) for 2 or 4 hours, and expression levels of MET, phosphorylated MET and NeuroD1 proteins were detected using Western Blotting. **I** The expression levels of MET and NeuroD1 protein in MET-overexpressing Panc-1 cells (MET-GFP) and control Panc-1 cells (GFP) were detected by Western Blotting; similarly, in MET-knockdown (sh#1, sh#2) and control Panc-1 cells (GFP), the expression levels of MET and NeuroD1 protein were detected by Western Blotting. **J** Neuropathiazol may influence the downstream signaling pathway by modulating the phosphorylation of the MET protein, thereby upregulating the expression of NeuroD1, as depicted in the model diagram. **K** Panc-1 cells were treated with PBS or Neuropathiazol (0.5µM or 2µM) for 4 hours, and the expression levels of PI3K, phosphorylated PI3K, AKT, phosphorylated AKT, and NeuroD1 proteins were detected by Western Blotting. **L** Panc-1 cells were treated with PBS or PI3K inhibitor LY294002 (0.5µM or 1µM), and the expression levels of PI3K, phosphorylated PI3K, AKT, phosphorylated AKT, and NeuroD1 proteins were detected by Western Blotting

We used the known 2D structure of Neuropathiazol and MOE software to generate a preliminary 3D structure for the compound (Fig. 6A). Hydrogen atoms were added to the structure using the Wash function under the Amber10-EHT force field. The energy of the 3D structure was minimized with rigid solvent water, using an optimal gradient root mean square (RMS) cutoff of 0.1 kcal/mol/Å. We then utilized PharmMapper (with a Z-score > 1), SwissTargetPrediction (with a Probability > 0), and the GalaxySagittarius method (with a docking score < −23.5) to predict the potential targets of Neuropathiazol. Table S4 shows the details used in target fishing. Collectively, these methods revealed a total of 175 possible targets for Neuropathiazol. Simultaneously, we gathered known targets for pancreatic cancer using GeneCards v5.15 (https://www.genecards.org/), with “pancreatic cancer” as the search term. By applying a score threshold of greater than 30, we identified 336 targets that were strongly associated with pancreatic cancer. Through intersection analysis of the 175 targets obtained via database screening and the 336 targets identified via manual screening, we identified 15 targets that were identified by both methods and were confirmed by MOE software as capable of docking with Neuropathiazol (Fig. 6, B and C). MOE software was used to evaluate the interaction energy between proteins and Neuropathiazol; the scoring value is presented as the S value. A lower S value indicates a stronger affinity between the two entities. Among the 15 potential targets identified, we determined that the MET protein (also known as hepatocyte growth factor receptor, HGFR) was the only membrane protein, and it appeared to be the most likely target of Neuropathiazol.

To further investigate whether Neuropathiazol stably binds MET, we conducted 15-ns molecular dynamics simulations. RMSD analysis of the simulation results revealed that the system began to converge after 2 ns, with the structure reaching equilibrium at approximately 5 ns. The overall RMSD value fluctuated around 2.5 Å (Fig. 6D). We subsequently selected structures from the 5–15 ns timeframe for MM-GBSA binding free energy analysis. The results indicated that the binding free energy stabilized around − 50 kcal/mol, with an average value of −46.60 kcal/mol. Notably, the average binding free energy between 8 and 15 ns was − 48.96 kcal/mol (Fig. 6E). This indicates that there is a strong interaction between them. Through computer simulations of the conformation of the Neuropathiazol-MET protein complex, we discovered that Neuropathiazol binds to the MET protein via a hydrogen bond with His-1202 (Fig. 6F). Specifically, Neuropathiazol binds to the MET protein between amino acid residues 1127 and 1260. Surprisingly, this sequence is located within the phosphorylated region of the MET protein. To further investigate this interaction, we employed surface plasmon resonance (SPR) technology to determine the affinity between the MET protein and Neuropathiazol. The results revealed a dissociation constant (KD) of 12.5e-5, indicating very strong affinity between Neuropathiazol and the MET protein (Fig. 6G). These findings indicate that the MET protein is likely a target of Neuropathiazol. The Neuropathiazol and MET proteins have strong affinities for each other, and Neuropathiazol may exert its effects by binding to the MET protein and modulating its phosphorylation.

We subsequently treated Panc-1 cells with 0.5 µM or 2 µM Neuropathiazol for 2–4 h, using PBS as a control. Proteins were collected, and the levels of expression of MET and phosphorylated MET were assessed via Western Blotting. We observed that Neuropathiazol inhibited the phosphorylation of the MET protein in Panc-1 cells, which was followed by an upregulation of NeuroD1 expression (Fig. 6H). Furthermore, by constructing MET-overexpressing Panc-1 cells, we observed that the expression of NeuroD1 was downregulated compared to control Panc-1cells; similarly, NeuroD1 expression was increased in MET-knockdown Panc-1 cells (Fig. 6I). Collectively, these findings support the conclusion that Neuropathiazol upregulates NeuroD1 expression by interfering with the phosphorylation of MET protein and subsequently modulating downstream signaling pathways (Fig. 6J). Upon reviewing the literature, we found that previous studies had demonstrated that MET phosphorylation activates the PI3K-AKT signaling pathways [50, 51]. Therefore, we examined the levels of expression of PI3K, phosphorylated PI3K, AKT, phosphorylated AKT, and NeuroD1 in the cells after Neuropathiazol treatment using Western Blotting. We discovered that Neuropathiazol treatment inhibited the phosphorylation of PI3K and AKT while increasing NeuroD1 expression (Fig. 6K). Likewise, by using LY294002, a chemical inhibitor of PI3K, blocking the activation of the PI3K-AKT signaling pathway also induced the upregulation of NeuroD1 expression [52] (Fig. 6L). These results suggest that Neuropathiazol binds to the MET protein, inhibits its phosphorylation, and thereby suppresses the activation of the downstream PI3K-AKT signaling pathway, ultimately leading to upregulation of NeuroD1 expression.

### NeuroD1 interacts with Neurog3 to inhibit the proliferation and transdifferentiation of pancreatic cancer cells

**Fig. 7:**
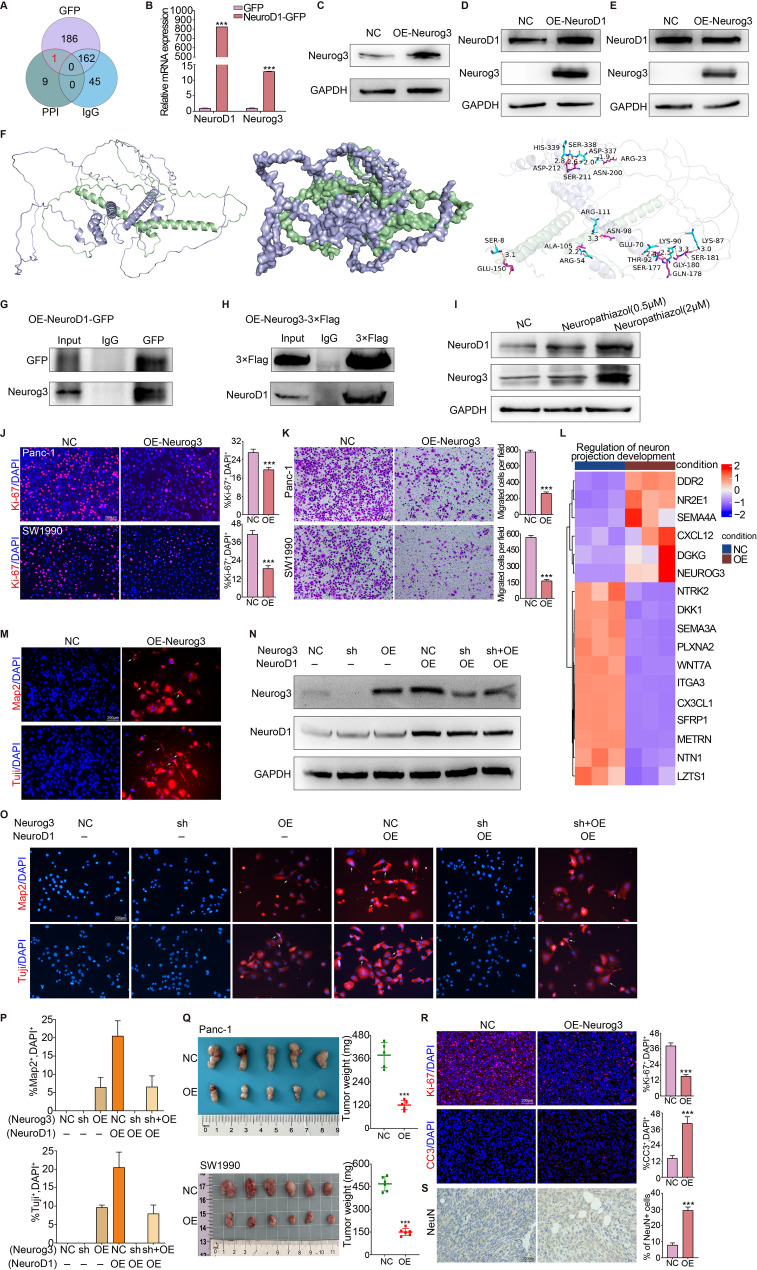
NeuroD1 interacts with Neurog3 to inhibit the proliferation and transdifferentiation of pancreatic cancer. **A** Neurog3 was identified as a downstream protein of NeuroD1 through three parts of data: (1) proteins interacting with normal IgG in the Co-IP experiment (in blue color); (2) proteins interacting with NeuroD1-GFP in the Co-IP experiment (in purple color); (3) proteins interacting with NeuroD1 in the Protein-Protein Interaction (PPI) Network database (in green color). **B** The expression levels of NeuroD1 and Neurog3 were assessed in NeuroD1-overexpressing Panc-1 cells using RT-qPCR. **C** Western Blotting was performed to confirmed the successful construction of the stable Panc-1 cell lines overexpressing Neurog3. **D** The expressions of NeuroD1 and Neurog3 in NeuroD1-overexpressing Panc-1 cells were detected by Western Blotting. **E** The expressions of NeuroD1 and Neurog3 in Neurog3-overexpressing Panc-1 cells were detected by Western Blotting. **F** The NeuroD1 (in purple)-Neurog3 (in green) protein docking was analyzed using the UniProtKB database. **G** In CO-IP experiments, Neurog3 was successfully precipitated using antibodies specific to NeuroD1-GFP in GFP-labeled NeuroD1-overexpressing Panc-1 cells (OE-NeuroD1-GFP). **H** In CO-IP experiment, NeuroD1 was successfully precipitated by using antibodies specific to Neurog3-3×Flag in 3×flag-labeled Neurog3 overexpressed Panc-1 cells (OE-Neurog3-3×Flag). **I** Panc-1 cells were treated with PBS or Neuropathiazol (0.5µM or 2µM) for 48 h, and the expression levels of NeuroD1 and Neurog3 were detected by Western Blotting. **J** In control (NC), Neurog3-overexpressing (OE-Neurog3) pancreatic cancer cells (Panc-1/SW1990), IF staining was used to mark Ki-67 protein (in red color) to label proliferating cells. Use DAPI to reverse stain the nucleus (blue). Nuclei were counterstained with DAPI (in blue color). Scale bar: 200 μm. The percentage of Ki-67^+^ cells was quantified. **K** The migration ability of NC and OE-Neurog3 pancreatic cancer cells (Panc-1/SW1990) was evaluated by Transwell assay, and the number of migrated cells per field was counted. Scale bar: 200 μm. **L** NC and OE-Neurog3 Panc-1 cells were collected for transcriptome sequencing and subsequent analysis. A large number of differentially expressed genes, including Neurog3, were significantly enriched in signaling pathways of the regulation of neuronal projection development. **M** IF staining of Map2 and Tuj1 proteins was performed in NC and OE-Neurog3 Panc-1 cells to observe the morphology of cytoskeleton. Nuclei were counterstained with DAPI (in blue color). Scale bar: 200 μm. **N** Panc-1 cells were used to establish negative control (NC), Neurog3-knockdown (sh), and Neurog3-overexpressing (OE) cell lines; in parallel, NeuroD1-overexpressing Panc-1 cells were used to establish the dual-regulatory model as control (NC), Neurog3-knockdown (sh), and Neurog3-rescue (sh + OE) cell lines. The expression levels of NeuroD1 and Neurog3 were detected by Western Blotting. **O** IF staining of Map2 and Tuj1 proteins was performed to observe the morphology of cytoskeleton. Nuclei were counterstained with DAPI (in blue color). Scale bar: 200 μm. **P** The percentage of Map2^+^ or Tuj1^+^ cells were quantified. **Q** Subcutaneous tumor models were constructed using NC and OE-Neurog3 pancreatic cancer cells (Panc-1/SW1990). On the 14th day of subcutaneous tumor construction, the tumor were harvested, photographed and the tumor weight was measured. **R** Subcutaneous tumor tissue (of Panc-1 cells) was taken for IF staining for Ki-67 and CC3 protein (in red color) to label proliferating and apoptotic cells. Nuclei were counterstained with DAPI (in blue color). **S** IHC staining for NeuN protein was performed to label the pancreatic cancer cell that have transdifferentiated into mature neurons. Scale: 200 μm. The percentage of Ki-67^+^ cells, CC3^+^ cells and NeuN^+^ cells were quantified. * *p* < 0.05, ** *p* < 0.01, *** *p* < 0.001

Next, we sought to identify downstream proteins that interact with NeuroD1. We constructed GFP-labeled NeuroD1-overexpressing Panc-1 cells (OE-NeuroD1-GFP) and performed Co-IP coupled with mass spectrometry. The results are shown in Table S3. As shown in Fig. 7A, the Venn diagram included three parts of data: (1) proteins that interacted with normal IgG in the Co-IP experiment (in blue color); (2) proteins that interacted with NeuroD1-GFP in the Co-IP experiment (in purple color); and (3) proteins that were shown in the Protein-Protein Interaction (PPI) Network database to interact with NeuroD1 (in green color). Through this comprehensive analysis, we ultimately identified Neurogenin 3 (Neurog3) as a downstream protein that interacts with NeuroD1. Neurog3 is a key transcription factor belonging to the basic helix‒loop‒helix (bHLH) family and has been previously demonstrated to activate gene transcription in endocrine precursor cells. It plays a crucial role in the formation of endocrine cell lineages in the pancreas and intestine [53, 54]. To investigate the relationship between NeuroD1 and Neurog3, we initially assessed their expression levels in Panc-1 cells overexpressing NeuroD1 (OE-NeuroD1-GFP) using RT-qPCR. Our findings revealed that as NeuroD1 expression increased, Neurog3 expression was correspondingly upregulated (Fig. 7B). Subsequently, we established Neurog3-overexpressing cell lines and confirmed the successful construction via Western Blotting (Fig. 7C). Further analysis of NeuroD1 and Neurog3 expression in these overexpressing cell lines demonstrated that NeuroD1 overexpression consistently led to elevated Neurog3 expression levels (Fig. 7D). However, Neurog3 overexpression did not in turn result in the upregulation of NeuroD1 (Fig. 7E). These findings indicate that there may be an interaction or a regulatory relationship between NeuroD1 and Neurog3 in which NeuroD1 modulates the expression of Neurog3.

To further elucidate the interaction between the NeuroD1 and Neurog3 proteins, we conducted protein‒protein docking analysis using the UniProtKB database. The protein-protein docking results indicated a strong binding affinity between NeuroD1 and Neurog3, with a binding free energy of −16.6 kcal/mol. Figure 7F, in which the Neurog3 protein depicted in green and the NeuroD1 protein is shown in purple, illustrates the overall docking configuration. The surface model revealed that the interaction between the NeuroD1 and Neurog3 proteins is stabilized through extensive hydrogen bonding at their contact surfaces, thereby increasing the overall stability of the protein complex. Detailed analysis of the key amino acid residues involved revealed that hydrogen bonds are formed at multiple contact points between the key residues, further reinforcing the stability of the binding. Detailed information on the protein‒protein docking of NeuroD1 and Neurog3 are provided in Table S5. Overall, the protein-protein docking analysis demonstrated robust surface interactions between NeuroD1 and Neurog3, characterized by a highly stable binding energy. This strong and stable binding suggests that the interaction between these two proteins reached an optimal state and provides insight into their potential functional relationship.

To confirm the physical interaction between NeuroD1 and Neurog3, we performed Co-IP experiments using GFP-labeled NeuroD1-overexpressing Panc-1 cells (OE-NeuroD1-GFP) and 3×Flag-labeled Neurog3-overexpressing Panc-1 cells (OE-Neurog3-3×Flag). The results showed that Neurog3 could be effectively precipitated by an antibody specific to NeuroD1-GFP (Fig. 7G), whereas NeuroD1 could be successfully precipitated by an antibody specific to Neurog3-3×Flag (Fig. 7E). These findings provide further evidence for a direct interaction between NeuroD1 and Neurog3. Additionally, treatment with Neuropathiazol led to the concurrent upregulation of both NeuroD1 and Neurog3 levels in Panc-1 cells (Fig. 7I).

We subsequently investigated the effects of Neurog3 overexpression on the proliferation and migration abilities of Panc-1 and SW1990 cells. We found that Neurog3 overexpression significantly inhibited both the proliferation (Fig. 7J) and the migration (Fig. 7K) of pancreatic cancer cells. To further elucidate the mechanisms underlying these effects, we performed RNA sequencing and analyzed DEGs between control Panc-1 cells and Neurog3-overexpressing Panc-1 cells. The analysis revealed that the DEGs were significantly enriched in signaling pathways associated with the regulation of neuronal projection development (Fig. 7L). These findings suggest that Neurog3 may play a role in promoting the transformation of pancreatic cancer cells into cells with neuron-like phenotypes. Consistent with this, IF staining for Map2 and Tuj1 in Neurog3-overexpressing cells revealed that Neurog3 upregulated the transdifferentiation of pancreatic cancer cells into cells with neuron-like phenotypes (Fig. 7M).

We established control, Neurog3-knockdown, and Neurog3-overexpressing cell lines using Panc-1 cells. In parallel, we generated a dual-regulatory model in NeuroD1-overexpressing Panc-1 cells with control, Neurog3-knockdown, and Neurog3-rescue groups (Fig. 7N). IF staining for Map2 and Tuj1 revealed that in pancreatic cancer cells with low NeuroD1 expression, neither the control nor Neurog3-knockdown groups exhibited transition to a neuron-like phenotype, whereas Neurog3 overexpression partially induced this transition (Fig. 7O). In cells with high NeuroD1 expression, Neurog3 knockdown suppressed the neuron-like transition, and this suppression was rescued by restoring Neurog3 expression. Among the six cell groups, the Panc-1 cells with high NeuroD1 expression and no Neurog3 knockdown exhibited the highest proportion of cells that underwent neuron-like phenotypic transition. As quantified in Fig. 7P, the proportion of transdifferentiated cells in each group reveals that the transdifferentiation-inducing effect of NeuroD1 is dependent on Neurog3. However, in terms of inductive efficacy, NeuroD1 exhibits a significantly higher capacity than Neurog3 to induce pancreatic cancer cells to transdifferentiate into neuron-like phenotypes.

We established subcutaneous tumor models in mice using Panc-1 and SW1990 cells and used these models to investigate the effects of Neurog3 overexpression on the progression of pancreatic cancer in vivo. We found that Neurog3 overexpression significantly inhibited the in vivo progression of pancreatic cancer (Fig. 7Q). Subcutaneous tumor tissues were collected and subjected to IF and IHC staining for the Ki-67, CC3 and NeuN proteins. The results showed that Neurog3 overexpression markedly reduced the proliferative capacity of pancreatic cancer cells while significantly increasing apoptosis of the cells (Fig. 7R). The proportion of cells expressing neuronal markers was also significantly increased, indicating that Neurog3 induces the phenotypic transformation of pancreatic cancer cells into neuron-like cells (Fig. 7S). In summary, our findings indicate that NeuroD1 interacts with Neurog3, and suppresses the proliferation of pancreatic cancer cells both in vitro and in vivo. NeuroD1 and Neurog3 induce the transdifferentiation of pancreatic cancer cells into cells with neuron-like phenotypes.

### The MET-NeuroD1-Neurog3 axis suppresses tumor progression in pancreatic cancer

**Fig. 8:**
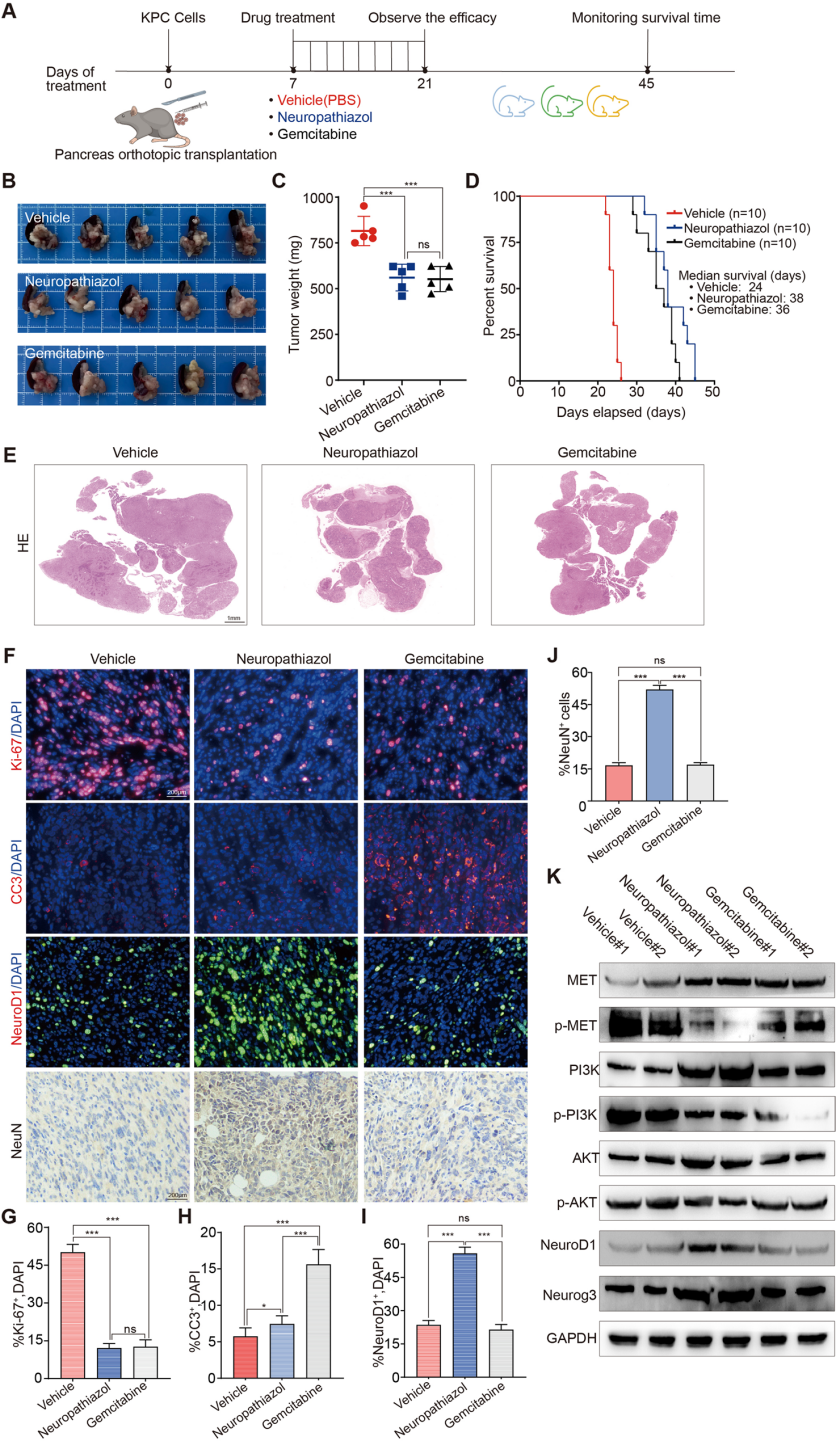
MET-NeuroD1-Neurog3 axis suppress tumor progression in pancreatic cancer. **A-K** Pancreatic cancer KPC cells were isolated from the spontaneously developed pancreatic ductal adenocarcinoma (PDAC) of LSL-KRas^G12D/+^-p53^R172H/+^-Pdx1-Cre (KPC) transgenic. Orthotopic pancreatic transplantation tumors were constructed by injecting KPC cells into the pancreas of mice. On the 7th day after tumor initiation, the mice were randomly assigned into three groups and treated with intraperitoneal injections of vehicle (PBS), Neuropathiazol (50 mg/kg), and gemcitabine (20 mg/kg), respectively, once every two days for a total of two weeks. Another cohort of mice underwent the same tumor induction and treatment regimen but continued to receive the assigned treatments until the mice succumbed to the disease. **A** The flow chart of the construction of orthotopic transplantation tumor models. **B** On day 14 after administration, tumors were collected and photographed. **C** Tumor weight was measured and statistically analyzed for each group. **D** Survival time was recorded and statistically analyzed for mice in each group. **E** H&E staining of mouse tumor tissue. **F** Orthotopic transplantation tumors were collected, IF staining for Ki-67 and CC3 proteins (in red color) was performed to label proliferating and apoptotic cells. IF staining for NeuroD1 protein (in green color) was performed to observe the expression level. Nuclei were counterstained with DAPI (in blue color). IHC staining for NeuN protein was performed to label the pancreatic cancer cell that have transdifferentiated into mature neurons. Scale: 200 μm. **G** The percentage of Ki-67^+^ cells were quantified. **H** The percentage of CC3^+^ cells were quantified. **I** The percentage of NeuroD1^+^ cells were quantified. **J** The percentage of NeuN^+^ cells were quantified. **K** The results showed that Neuropathiazol inhibited the phosphorylation of MET protein and the PI3K-AKT signaling pathway, upregulating the expression levels of NeuroD1 and Neurog3 protein detected by western blotting

We isolated pancreatic cancer cells from spontaneous tumors of KPC transgenic mice and established orthotopic pancreatic transplantation tumors. The mice were randomly assigned to three groups, in which the animals received intraperitoneal injections of vehicle (PBS), Neuropathiazol (50 mg/kg), or gemcitabine (20 mg/kg) every two days for two weeks beginning on day 7 after tumor implantation. After two weeks, the orthotopic pancreatic tumors were harvested, photographed, and weighed. Another cohort of mice underwent tumor implantation but received continuous administration of vehicle, Neuropathiazol, or gemcitabine until they died. Figure 8A shows a diagram of the experimental workflow. We found that Neuropathiazol treatment significantly inhibited pancreatic tumor growth (Fig. 8B and C) and extended the survival time of the mice (Fig. 8D), demonstrating a therapeutic effect comparable to that of gemcitabine.

Mouse plasma samples were collected at 0.083, 0.25, 0.5, 1, 2, 4, 6, 8, and 24 h following Neuropathiazol administration, and the concentration of Neuropathiazol was quantified via high-performance liquid chromatography-tandem mass spectrometry (LC-MS/MS). As illustrated in Figure S4A, Neuropathiazol reached its peak concentration in mouse plasma as early as 0.083 h post-administration, after which the concentration gradually decreased and became barely detectable by 8 h. To investigate its distribution in tissues, the concentrations of Neuropathiazol in mouse plasma, heart, liver, kidneys, lungs, and spleen were quantified at the peak time point (0.083 h) and two subsequent time points (0.25 h and 0.5 h) (Fig. S4B). The results showed that Neuropathiazol was well absorbed by the tissues, and its concentration gradually decreased after the peak time point. Detailed pharmacokinetic data, the in vivo dose-response curve, and tissue distribution data are presented in Table S6. The pharmacokinetic data demonstrate that Neuropathiazol exerts its effects with a rapid onset of action and is readily cleared from the body without accumulation. Moreover, continuous monitoring of the weight changes of the mice in each group during the treatment period revealed that treatment with Neuropathiazol did not significantly alter body weight, whereas gemcitabine treatment led to substantial weight loss (Fig. S4C). We also collected heart, liver, kidneys, lungs, and spleen tissues from the mice in each group and subjected them to H&E staining. The results indicated that Neuropathiazol did not cause significant organ damage (Fig. S4D). Collectively, these findings indicate that Neuropathiazol exerts its effects with a rapid onset of action and is readily cleared from the body without accumulation. Furthermore, it exhibits low drug toxicity and favorable safety profiles.

H&E staining of tumor tissues from the animals confirmed the successful establishment of orthotopic pancreatic graft tumors (Fig. 8E). The tumor tissues were subjected to IF staining for Ki-67, CC3, and NeuroD1. The results indicated that both Neuropathiazol and gemcitabine significantly inhibited the proliferation of pancreatic cancer cells (Fig. 8F, G) and promoted apoptosis of the cells (Fig. 8F, H). Neuropathiazol treatment also upregulated the expression of NeuroD1 in pancreatic cancer cells (Fig. 8F, I). IHC staining for the NeuN protein revealed that Neuropathiazol increased the proportion of NeuN-positive cells in pancreatic cancer tissues (Fig. 8F, J), suggesting that Neuropathiazol induces the transdifferentiation of some pancreatic cancer cells into neuron-like cells. This effect was not observed after gemcitabine treatment. On the basis of these results, we propose that gemcitabine exerts its antitumor effects through cytotoxicity, leading to the death of pancreatic cancer cells. In contrast, Neuropathiazol inhibits tumor progression by causing pancreatic cancer cells to adopt a more differentiated, neuron-like, benign phenotype. We further examined the expression and phosphorylation of the MET, PI3K, and AKT proteins in tumor tissues treated with Neuropathiazol or gemcitabine via Western Blotting. The results showed that Neuropathiazol inhibited phosphorylation of the MET protein and the activity of the PI3K-AKT signaling pathway, thereby increasing the expression levels of NeuroD1 and Neurog3 (Fig. 8K). However, gemcitabine did not alter the levels of expression of these proteins. In summary, our findings demonstrate that Neuropathiazol inhibits pancreatic cancer progression via the MET-NeuroD1-Neurog3 axis.

## Discussion

Pancreatic cancer is highly lethal, with a five-year survival rate of only approximately 10%, and poses serious health threats and significant economic and social burdens [55]. It is currently the sixth leading cause of cancer-related death worldwide, and its incidence is on the rise; it is projected to become the second leading cause of cancer death in Western countries [1, 2]. Surgery remains the only potentially curative treatment for pancreatic cancer. However, owing to disease progression, only 15–20% of patients are eligible for surgery at the time of diagnosis. Chemotherapy serves as a crucial adjuvant therapy and the primary treatment for advanced pancreatic cancer. However, chemotherapeutic drugs have substantial side effects; their short-term toxicity often results in gastrointestinal reactions and local pain, and their long-term toxicity may cause bone marrow suppression and visceral damage [56–58]. Moreover, the widespread presence of drug resistance also limits the application of chemotherapeutic drugs. How to shrink tumors to the greatest extent while minimizing the toxic effects of drugs is an important issue that is worth exploring.

Our research revealed that the transcription factor NeuroD1 is able to induce the transdifferentiation of tumor cells into neuron-like cells. This indicates that NeuroD1 has the potential to transform cancer cells from a malignant to a benign biological. NeuroD1 is an alkaline bHLH transcription factor that is expressed primarily in pancreatic and nerve cells and plays a crucial role in the differentiation and maturation of endocrine cells. Previous studies have demonstrated that NeuroD1 has the ability to induce the transdifferentiation of microglia and astrocytes into neurons in mice [20, 21]. In tumors, NeuroD1 promotes the differentiation of medulloblastoma cells into mature neurons [15, 22]. However, these prior investigations into NeuroD1-induced transdifferentiation have been primarily confined to scenarios of neurological injury repair or neurogenic tumors. Our study is the first to introduce the NeuroD1-mediated transdifferentiation mechanism into solid tumors of the digestive system, breaking the previous limitation to the context of “neurogenesis or neurogenic tumors”. Notably, the specific mechanism by which NeuroD1 drives the transdifferentiation of pancreatic cancer cells differs from that in other tumor types. Pancreatic cancer is an extremely aggressive solid tumor of the digestive system of epithelial origin, exhibiting substantial differences in molecular characteristics and tumor microenvironment compared to neurogenic tumors and even other tumor types. For instance, following ischemic brain injury, overexpression of NeuroD1 in astrocytes enables this bHLH transcription factor to directly bind to the promoters and enhancers of neuron-related genes. By activating neuron-specific transcriptional programs and repressing glial gene expression, NeuroD1 drives the direct transdifferentiation of astrocytes into functional neurons. This process induces the direct reprogramming of non-neuronal cells (particularly astrocytes) into neurons, thereby facilitating brain function repair [21]. Similarly, in medulloblastoma, NeuroD1 promotes medulloblastoma cells to acquire mature neuronal characteristics, again through activating neuron-specific gene expression programs, ultimately leading to the loss of their tumor-initiating and proliferative capacities [22]. In contrast, our findings revealed that the role of NeuroD1 in pancreatic cancer is mediated through Neurog3. And the rescue experiments confirmed that Neurog3 is indispensable for the transdifferentiation of pancreatic cancer cells.

Through drug screening analysis and experiments, we identified Neuropathiazol, a small-molecule compound that potentially induces the specific upregulation of NeuroD1 expression in pancreatic cancer cells. Further mechanistic studies revealed that Neuropathiazol binds to the MET protein on the cell membrane and inhibits its phosphorylation, thereby suppressing the activation of the downstream PI3K-AKT signaling pathway and increasing NeuroD1 expression. While previous studies have established that NeuroD1 expression in medulloblastoma is epigenetically regulated, our research uncovers a distinct regulatory mechanism in pancreatic cancer, differing from that observed in neurogenic tumors [22]. Importantly, this finding providing insights for clinical translation. It suggests that upregulating NeuroD1 expression in pancreatic cancer cells via effective targeted drugs holds promise for inhibiting pancreatic cancer progression and offers new hope for pancreatic cancer treatment. Both previous studies and our present work indicate that pancreatic cancer cells retain a certain degree of plasticity, allowing their conversion to a neuron-like phenotype induced by neural transcription factors such as NeuroD1. We propose that NeuroD1-mediated neuronal transdifferentiation can significantly attenuate the malignant phenotype of pancreatic cancer. Transdifferentiated cells exhibit neuron-like characteristics and enter a state of terminal differentiation with loss of proliferative capacity, thereby permanently exiting the cell cycle and losing the ability for sustained division and invasion. Concurrently, in our in vivo models, we observed activation of the TNFα signaling pathway and an increased proportion of apoptotic cells (CC3-positive cells). These findings suggest that transdifferentiation not only inhibits proliferation signals but also triggers pro-apoptotic mechanisms. Against this backdrop, inducing neuronal transdifferentiation in pancreatic cancer cells demonstrates clear rationality and clinical translational value. Although our study has not yet identified such differentiated cells in single-cell sequencing data of pancreatic tumor cells, similar findings have been reported in other cancer types, including medulloblastoma. Thus, the strategy of inducing tumor cell transdifferentiation is expected to be applicable across a broad spectrum of cancers, encompassing both pancreatic cancer and medulloblastoma.

The impact of transdifferentiation on the tumor immune microenvironment also merits attention. The emergence of neuron-like cells may alter the secretion profile of cytokines and chemokines, thereby reshaping the infiltration pattern of immune cells. Alternatively, these neuron-like cells might express novel surface antigens or modify the presentation pattern of MHC molecules, which could enhance their recognition by T cells and render them more susceptible to immune surveillance. On the other hand, neural infiltration in pancreatic cancer is often accompanied by an immunosuppressive microenvironment; transdifferentiation may disrupt this interaction and restore anti-tumor immunity.

Nonetheless, this study still has certain limitations. Pancreatic cancer often exhibits perineural invasion, with active crosstalk between tumor cells and the neural microenvironment. Consequently, the strategy of inducing tumor cell transdifferentiation into neurons may also carry potential risks. It cannot be ruled out that some transdifferentiated cells might acquire “neuronal functions”, which could reshape the tumor microenvironment. However, compared to tumor cells, neuron-like cells may be more susceptible to cytotoxic drugs, thereby enhancing sensitivity to chemotherapy. Our study has confirmed this phenomenon when the transdifferentiation-inducing agent Neuropathiazol was combined with chemotherapeutic drugs, a more significant reduction in tumor volume was observed. Clinically, neuroendocrine tumors have a much better prognosis than pancreatic cancer, which to some extent validates the feasibility of the transdifferentiation strategy. On the other hand, the long-term fate and phenotypic stability of transdifferentiated neuron-like cells in vivo remain unclear, and further verification is required to determine whether these cells can revert to their original state under the influence of the microenvironment. Additionally, transdifferentiated neuron-like tumor cells may form abnormal connections with the host neural network, potentially leading to aberrant neural signaling or pain. Future studies should further conduct lineage tracing experiments using the Cre-loxP fluorescent reporter system to dynamically monitor the long-term fate of transdifferentiated cells in animal models, thereby verifying their phenotypic stability and irreversibility in vivo. Concurrently, integrating single-cell RNA sequencing will enable systematic analysis of gene expression profiles before and after transdifferentiation, facilitating the identification of key regulatory factors that maintain the neuronal state and the discovery of novel potential targets. Furthermore, since transdifferentiation may exert profound impacts on the tumor immune microenvironment, it is necessary to utilize single-cell and spatial omics technologies to evaluate the infiltration characteristics and functional states of immune cells pre- and post-transdifferentiation. This will also allow for further exploration of their synergistic effects with immune checkpoint inhibitors. In terms of safety, neurological function monitoring and pain behavior assessments should be performed in animal models to identify potential adverse neural connections and functional abnormalities. These efforts will provide a solid foundation for the preclinical validation and clinical safety evaluation of the transdifferentiation strategy. For translational application, the induction conditions need to be strictly regulated to minimize the risk of abnormal connections between transdifferentiated cells and the host neural network. Building on this, combination therapeutic strategies can be explored. For instance, considering the long-term in vivo stability of transdifferentiated cells and the potential off-target effects of Neuropathiazol in non-tumor tissues, we explored a strategy combining Neuropathiazol with cytotoxic chemotherapeutic drugs. This approach aims to non-selectively eliminate residual tumor cells, thereby further enhancing the efficacy and translational potential of transdifferentiation-based therapy. Notably, this combined strategy has demonstrated superior therapeutic effects compared to single-agent treatment in the present study. Additionally, the combined use of transdifferentiation inducers with immune checkpoint inhibitors or anti-neurotrophic factor drugs may enhance anti-tumor efficacy while reducing potential adverse events. Systematic preclinical studies, including target validation, dose optimization, and long-term safety monitoring, will lay a solid foundation for the clinical translation of transdifferentiation-based therapy for pancreatic cancer.

## Conclusions

**Fig. 9:**
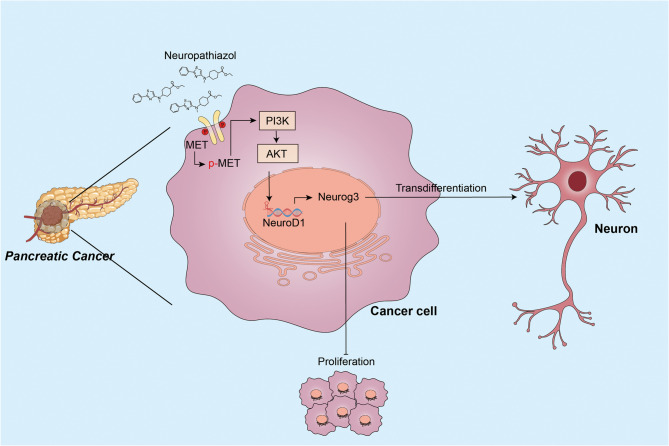
Graphical diagram of Neurogenic inducers inhibit the proliferation of pancreatic cancer by promoting tumor cell transdifferentiation. NeuroD1 overexpression induces the transdifferentiation of pancreatic cancer cells into neurons, thereby curbing tumor cell proliferation and hindering pancreatic cancer progression. Neurogenic inducer Neuropathiazol can elevate NeuroD1 expression by binding to the MET protein and inhibiting the activation of the PI3k/Akt signaling pathway. Neurog3 is a downstream protein that interacts with NeuroD1

Our study showed that NeuroD1 is a protective factor in pancreatic cancer, with high NeuroD1 expression correlating with improved patient outcomes. Through a comprehensive series of in vitro and in vivo experiments, we discovered that NeuroD1 overexpression can induce the transdifferentiation of pancreatic cancer cells into neurons, thereby curbing tumor cell proliferation and migration and hindering pancreatic cancer progression in vivo. Through drug screening and computer simulation, we demonstrated that the neuronal inducer Neuropathiazol increases NeuroD1 expression by binding to the MET protein and inhibiting the activation of the PI3k/Akt signaling pathway. Concurrently, we identified Neurog3 as a downstream protein that interacts with NeuroD1 (Fig. 9). Our research suggests that NeuroD1 may represent a potential therapeutic target for pancreatic cancer, inducing the reversal of the malignant phenotype of pancreatic tumor cells via transdifferentiation into neurons through gene editing or the use of differentiation-inducing agents. Neuropathiazol is anticipated to be the subject of further investigation as a potential differentiation-inducing agent, as it shows excellent efficacy along with high efficiency and low toxicity. Our study provides novel perspectives for pancreatic cancer treatment.

## Supplementary Information

Supplementary Material 1.

Supplementary Material 2.

Supplementary Material 3.

Supplementary Material 4.

Supplementary Material 5.

Supplementary Material 6.

Supplementary Material 7: Fig.S1 NeuroD1 expression is positively correlated with pancreatic cancer differentiation. (A) The true differentiation levels of 4 patients recorded in the clinical phenotype data of TCGA (PAAD) datasets were largely consistent with the predicted trends of the singlescore differentiation score. (B) Representative images of IHC staining in NeuroD1 high expression group and NeuroD1 low expression group.

Supplementary Material 8: Fig.S2 Neuropathiazol induces the upregulation of NeuroD1 expression with low cytotoxicity. (A) The half-maximal inhibitory concentration (IC50) of UNC1999 on Panc-1 cells. (B) The IC50 of UNC1999 on SW1990 cells. (C) The UNC1999 does not alter the expression level of NeuroD1 in Panc-1 cells. (D) The UNC1999 does not alter the expression level of NeuroD1 in SW1990 cells. (E) Panc-1 cells were treated with various compounds and commonly used first-line chemotherapy drugs at a concentration of 2µM for 48 hours, and the activity of the NeuroD1 promoter was evaluated by detecting the relative luciferase activity. (F) SW1990 cells were treated with various compounds and commonly used first-line chemotherapy drugs at a concentration of 2µM for 48 hours, and the activity of the NeuroD1 promoter was evaluated by detecting the relative luciferase activity. (G) Panc-1 cells were treated with various compounds and commonly used first-line chemotherapy drugs at a concentration of 2µM for 48 hours, and the expression level of NeuroD1 was detected by qPCR. (H) SW1990 cells were treated with various compounds and commonly used first-line chemotherapy drugs at a concentration of 2µM for 48 hours, and the expression level of NeuroD1 was detected by qPCR. (I) The IC50 of various compounds and commonly used first-line chemotherapy drugs on Panc-1 cells. (J) The IC50 of various compounds and commonly used first-line chemotherapy drugs on SW1990 cells.

Supplementary Material 9: Fig.S3 Combination with gemcitabine enhances the antitumor effect of Neuropathiazol in vivo. A subcutaneous tumor model was established in mice using Panc-1 cells. When tumor volumes reached approximately 200 mm³, mice were randomly divided into four groups and treated with a control vehicle, gemcitabine (20 mg/kg), Neuropathiazol (50 mg/kg), or Neuropathiazol (50 mg/kg) combined with gemcitabine (20 mg/kg). All treatments were administered via intraperitoneal injection every two days for two weeks. (A) Representative images of tumors dissected from each group after the treatment period. (B) Quantification of tumor volumes from each group at the end of the experiment. (C) Representative IF staining images for the proliferation marker Ki-67 in tumor tissues. Scale bar, 200 μm. (D) The percentage of Ki-67^+^ cells.

Supplementary Material 10: Fig.S4 Pharmacokinetic characteristics and safety evaluation of Neuropathiazol in vivo. (A) At 0.083, 0.25, 0.5, 1, 2, 4, 6, 8, and 24 hours after intraperitoneal injection of Neuropathiazol at a dose of 50 mg/kg, the concentrations of Neuropathiazol in mouse plasma. (B) At 0.083, 0.25, and 0.5 hours after intraperitoneal injection of Neuropathiazol at a dose of 50 mg/kg, the concentrations of Neuropathiazol in mouse plasma, heart, liver, kidney, lung, and spleen tissues. (C) In the experiment of orthotopic pancreatic transplantation tumors in mice, the change of body weight was monitored every 2 days during the administration of the drug, as shown in the line chart. (D) In the experiment of orthotopic pancreatic transplantation tumors in mice, the main internal organs were collected for H&E staining after administration to observe the visceral toxicity.

## Data Availability

All data generated or analyzed during this study are included in this published article and its supplementary information files. Source data are provided in this paper.

## Abbreviations

PDAC: Pancreatic ductal adenocarcinoma
GO: Gene Ontology
KEGG: Kyoto Encyclopedia of Genes and Genomes
GSEA: Gene Set Enrichment Ananlysis
GEO: Gene Expression Omnibus
TCGA: The Cancer Genome Atlas
GOPB: Gene Ontology Biological Process
shRNA: Short hairpin RNA
WB: Western blotting
RT-qPCR: Reverse transcription quantitative polymerase chain reaction
PBS: Phosphate-buffered salin
LC-MS/MS: Liquid chromatography-tandem mass spectrometry
IHC: Immunohistochemistry
Neurod1: Neurogenic differentiation 1
Neurog3: Neurogenin 3
Map2: Microtubule-associated protein 2
CC3: Cleved Caspase-3
Tuj1: Neuronal Class III β-Tubulin
FBS: Fetal Bovine Serum
