# Supplementary material for: Neurogenic inducers inhibit the proliferation of pancreatic cancer by promoting tumor cell transdifferentiation
Source: J Exp Clin Cancer Res. 2025 Nov 12;44:304. doi: 10.1186/s13046-025-03563-9 (PMC12613626; doi:10.1186/s13046-025-03563-9)
Supplement: Supplementary file 2 — Supplementary Material 2. [file 13046_2025_3563_MOESM2_ESM.docx]

TGGGTGAATTATTTTATCCTCCCCTATGCGCTTTGTCAGCCAAGTGAATGTAGAAAAGCAAGCAAAACTCCAGAACCCCACAACGATTCACGCCACGCTTCGGTGGCACAACTCTTTTCTTCCCCTTTTAGATTAGTACTGTGCAGGGAAGGCTGCACCTGCTACCTTCAGACACCGGGGATCTGGCTAGGACCCTCTTCCTCTACCCCTACTCTCTCCTCCCGTCCCCACCCTCTCCCAGATGCACCCCAATCCAGCCTCTCAACCCTGTCGCTGTTTGCAAACTCGAGGGATGTTTAGTGCAATTGCCTTTAAATAAGCAATTCACGTAAGAGGTATTTATTGCAGAGGGTTTCAGGTCTGGGAGGGGGTAAGGAGTTAGTTAATGAAGGATTAGAGGCAAAGCTGGGAAAGAACGAGTAGACAGGCCAGTTTCTTTCTTTCTTTTTAAAATCTATTATTCTGGAAAACCCTTGTTCGAAAAGAGTAAAGAATTGCTATTTGGGCAACAATATGGCTCCTTCAGAACTGGTCGAGAATTTCTGTGGTCTTTGCTTGACCTTCAAGTTGGGAGTTTCAAATCAGAGGCTTTGAAGTCTGGAGGAGTTCGCAGCCATTAATCCTTCCGGGGTTGTGCTAAAGGCGTTTCTAGAAAGAGATCGGAAAGACCCGTTCAGCACAGGCCCATTGCATCTTCTCTTTGAAAACGCTTGTCTGAATCAAGTATCTTATACAGACCCGAGACTGAGGGGAGCAAGAGCGCAGGTCCGGCTGTGGCGAGGTGTCCACCACCGTACTTGTGGTCAATGCCTGGACTAGAGAGCCGAGGCCGTCCAGGGCGGGCTGGCCAGGGAACCAGAAGCGTCCCTGGACTGGCTGAGTGAGGGTGCCCTTTCCAGCAACCCAGTGGCCATGGTTAAAAGGCAGAATACAACCGTCAAAGAAGTCCCCAGCTCCCACATAAGAGAGCGCAAAGGCCAATGGAACCAAGGCCTCCTCGCGAGAACTAGCTCGCTTTGAGGACAAGATCCTGGGGATGGAGGTGGGGACTGACGTAGTGAGAGGGTCTGGAGGAACAGTCGTAGCTGAAGGTCAGGACATGAAGAATTGCACGTATGGTAGGGATAGAATGGCAGGTACAACTTTTCTGTACTGAAGGAACTGGGAGAGGACGATCCGGTTAGGGAGGTTGGGGAACTAATCTCAACGCTGCGTTTACAGATGAAGCCGCTTTTATATGGCGTATATGTTTGCTTAGAGGGGCCGACGGAGATTAGGAGAAGCCATCCTTTGGCGCCAATGATCAAAGCGTCTGCCAAGGAGAAGAAGCCAAGGGATGGGCCTTTCAGAGAGGGCAAGGAGTCATGCTGCTCTGGATGCCAGTGTCAGGACAAGAAATCGAAAGGAGCGAGGACTCTTCACTGCGTGCCTCAGTCTCCCCGCTTCTGCCTCTTTCACCTCTGTCCTACTTCCGGCGCGAAAGCAGGCCACTCGCTCTGATCTAGACCTAGTTAACATTAGCTTTTCCCTTCCTTCCCTCAAATCCCCTCCCCCCTCTATCCCCGTCCCTTCTGCCGCCTGAAAGGGTTAATCTCTCCTGCGGGTAAAAACAGGTCCGCGGAGTCTCTAACTGGCGACAGATGGGCCACTTTCTTCTGGCCACAAAGGGGCCGGAATGGAGCGCTCCGCGGCATACAAATGGGCAGGTCACGTGGTTCCAGGCTCTTGGCTGGACCGGGAAGACCATATGGCGCATGCCGGGGAGGAAGGAGGAGGGGCGGGGGTAGGGGTGGAGGGTGAGGGGAGCGGTTGTCGGAGGAGGGCGGGAGACGAGCAAGGCGTGGGGAGAAGTGGGGAGGAGGGGAGAACGGGGAGCGCACAGCCTGGACGCGTGCGCAGGCGTCAGGCGCATAGACCTGCTAGCCCCTCAGCTAGCGGCCCCGCCCGCGCTTAGCATCACTAACTGGGCTATATAACCTGAGCGCCCGCGCGGCCACGAC
