# Supplementary figures and images for: Neurogenic inducers inhibit the proliferation of pancreatic cancer by promoting tumor cell transdifferentiation
Source: J Exp Clin Cancer Res. 2025 Nov 12;44:304. doi: 10.1186/s13046-025-03563-9 (PMC12613626; doi:10.1186/s13046-025-03563-9)

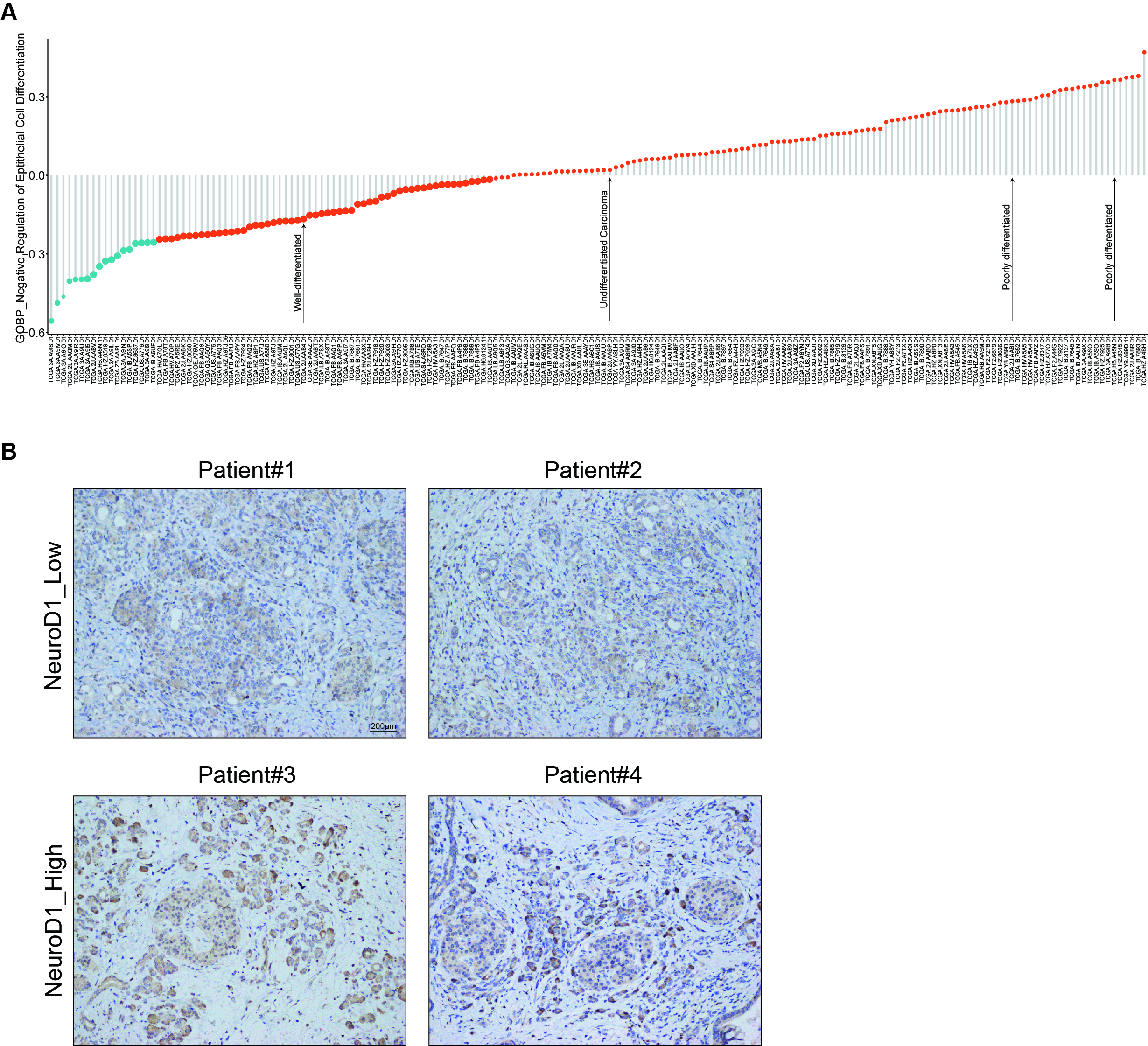

Supplement: Supplementary file 7 — Supplementary Material 7: Fig.S1 NeuroD1 expression is positively correlated with pancreatic cancer differentiation. (A) The true differentiation levels of 4 patients recorded in the clinical phenotype data of TCGA (PAAD) datasets were largely consistent with the predicted trends of the singlescore differentiation score. (B) Representative images of IHC staining in NeuroD1 high expression group and NeuroD1 low expression group. [file 13046_2025_3563_MOESM7_ESM.jpg]

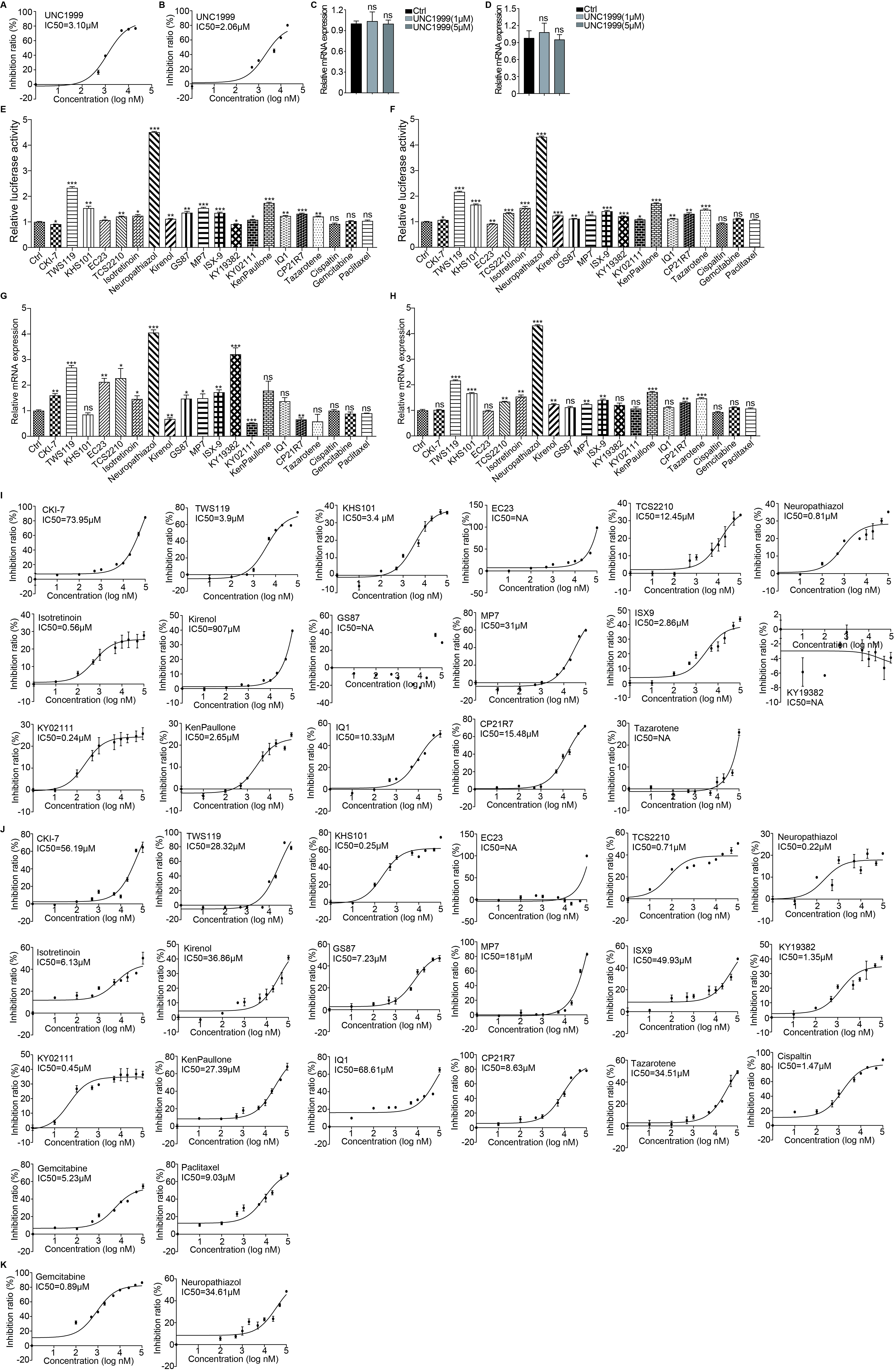

Supplement: Supplementary file 8 — Supplementary Material 8: Fig.S2 Neuropathiazol induces the upregulation of NeuroD1 expression with low cytotoxicity. (A) The half-maximal inhibitory concentration (IC50) of UNC1999 on Panc-1 cells. (B) The IC50 of UNC1999 on SW1990 cells. (C) The UNC1999 does not alter the expression level of NeuroD1 in Panc-1 cells. (D) The UNC1999 does not alter the expression level of NeuroD1 in SW1990 cells. (E) Panc-1 cells were treated with various compounds and commonly used first-line chemotherapy drugs at a concentration of 2µM for 48 hours, and the activity of the NeuroD1 promoter was evaluated by detecting the relative luciferase activity. (F) SW1990 cells were treated with various compounds and commonly used first-line chemotherapy drugs at a concentration of 2µM for 48 hours, and the activity of the NeuroD1 promoter was evaluated by detecting the relative luciferase activity. (G) Panc-1 cells were treated with various compounds and commonly used first-line chemotherapy drugs at a concentration of 2µM for 48 hours, and the expression level of NeuroD1 was detected by qPCR. (H) SW1990 cells were treated with various compounds and commonly used first-line chemotherapy drugs at a concentration of 2µM for 48 hours, and the expression level of NeuroD1 was detected by qPCR. (I) The IC50 of various compounds and commonly used first-line chemotherapy drugs on Panc-1 cells. (J) The IC50 of various compounds and commonly used first-line chemotherapy drugs on SW1990 cells. [file 13046_2025_3563_MOESM8_ESM.jpg]

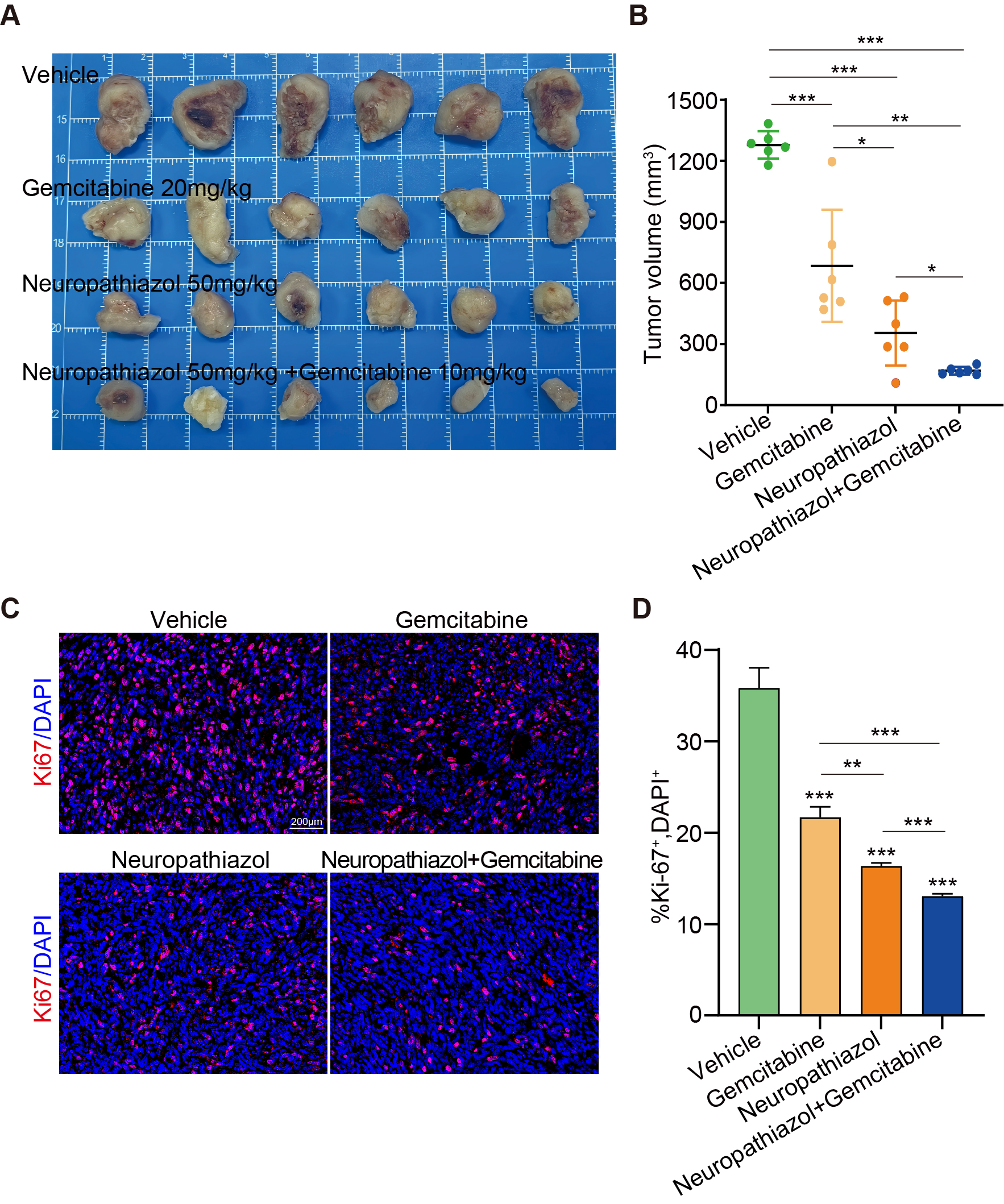

Supplement: Supplementary file 9 — Supplementary Material 9: Fig.S3 Combination with gemcitabine enhances the antitumor effect of Neuropathiazol in vivo. A subcutaneous tumor model was established in mice using Panc-1 cells. When tumor volumes reached approximately 200 mm³, mice were randomly divided into four groups and treated with a control vehicle, gemcitabine (20 mg/kg), Neuropathiazol (50 mg/kg), or Neuropathiazol (50 mg/kg) combined with gemcitabine (20 mg/kg). All treatments were administered via intraperitoneal injection every two days for two weeks. (A) Representative images of tumors dissected from each group after the treatment period. (B) Quantification of tumor volumes from each group at the end of the experiment. (C) Representative IF staining images for the proliferation marker Ki-67 in tumor tissues. Scale bar, 200 μm. (D) The percentage of Ki-67+ cells. [file 13046_2025_3563_MOESM9_ESM.jpg]

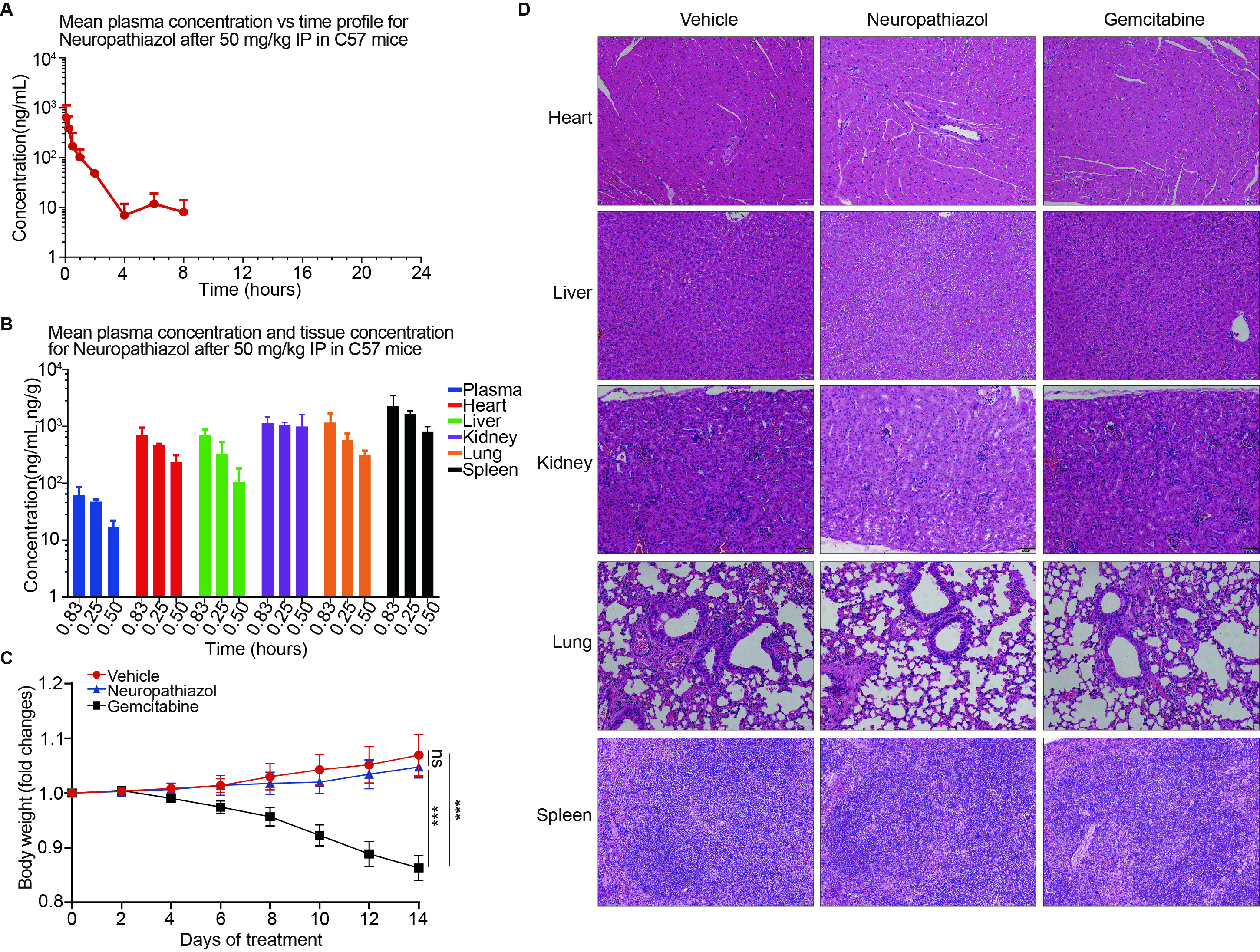

Supplement: Supplementary file 10 — Supplementary Material 10: Fig.S4 Pharmacokinetic characteristics and safety evaluation of Neuropathiazol in vivo. (A) At 0.083, 0.25, 0.5, 1, 2, 4, 6, 8, and 24 hours after intraperitoneal injection of Neuropathiazol at a dose of 50 mg/kg, the concentrations of Neuropathiazol in mouse plasma. (B) At 0.083, 0.25, and 0.5 hours after intraperitoneal injection of Neuropathiazol at a dose of 50 mg/kg, the concentrations of Neuropathiazol in mouse plasma, heart, liver, kidney, lung, and spleen tissues. (C) In the experiment of orthotopic pancreatic transplantation tumors in mice, the change of body weight was monitored every 2 days during the administration of the drug, as shown in the line chart. (D) In the experiment of orthotopic pancreatic transplantation tumors in mice, the main internal organs were collected for H&E staining after administration to observe the visceral toxicity. [file 13046_2025_3563_MOESM10_ESM.jpg]
