# Supplementary material for: Neurogenic inducers inhibit the proliferation of pancreatic cancer by promoting tumor cell transdifferentiation
Source: J Exp Clin Cancer Res. 2025 Nov 12;44:304. doi: 10.1186/s13046-025-03563-9 (PMC12613626; doi:10.1186/s13046-025-03563-9)
Supplement: Supplementary file 5 — Supplementary Material 5. [file 13046_2025_3563_MOESM5_ESM.zip › Table S6/The results of Protein-protein docking.doc]

Green：NEUROG3（Q9Y4Z2）

Purple：NEUROD1（Q13562）
